# Trazodone changed the polysomnographic sleep architecture in insomnia disorder: a systematic review and meta-analysis

**DOI:** 10.1038/s41598-022-18776-7

**Published:** 2022-08-24

**Authors:** Yongliang Zheng, Tian Lv, Jingjing Wu, Yumeng Lyu

**Affiliations:** 1 School of Rehabilitation Medicine, Jiangsu Vocational College of Medicine, Yancheng, China; 2 Department of Neurology, Zhuji Hospital Affiliated Shaoxing University, Shaoxing, China; 3 Department of Cardiology, Pudong New Area People’s Hospital, Shanghai, China

**Keywords:** Drug development, Sleep disorders

## Abstract

Trazodone has been widely prescribed for off-label use as a sleep aid. Identifying how trazodone impacts the performance of polysomnographic sleep architecture in insomnia disorder will provide additional data that can be used to guide clinical application. To assess the efficacy of trazodone in altering the polysomnographic sleep architecture in insomnia disorder so that sleep can be facilitated. PubMed, EMBASE, Web of Science, PsycINFO, Cochrane Library, Chinese Biomedical Literature Database (SinoMed), China National Knowledge Infrastructure, Wanfang Database, and the China Science and Technology Journal Database were searched for articles published between inception and June 2022. RCTs in patients with insomnia disorder applying trazodone in one arm of interventions at least 1 week, and reporting PSG parameters in the outcomes were eligible. RoB 2 was used to evaluate the risk of bias. The results of quality of evidence assessed by the Grading of Recommendations Assessment, Development and Evaluation (GRADE) approach. When *I*^2^ < 50%, the fixed effects model was used. When *I*^2^ ≥ 50%, the random effects model was used. The mean differences (MD) or standardized mean differences (SMD) and odds ratios (OR) with 95% confidence intervals (CIs) were estimated. Eleven randomized controlled trials were selected and participants were 466. Risk of bias was low in 5 trials (45.5%), and was moderate in 6 (54.5%). Compared with the control group, trazodone significantly increased total sleep time (TST, min) (MD = 39.88, 95% CI 14.44–65.32, *P* = 0.002) and non-rapid eye movement stage 3 (N3, mixed min and %) (SMD = 1.61, 95% CI 0.69–2.53, *P* = 0.0006); trazodone significantly decreased latency to onset of persistent sleep (LPS, min) (MD = − 19.30, 95% CI − 37.28 to − 1.32, *P* = 0.04), non-rapid eye movement stage 1 (N1, mixed min and %) (SMD = − 0.62, 95% CI − 1.13 to − 0.12, *P* = 0.02), the number of awakenings (NAs, including both arousal times and arousal index) (SMD = − 0.67, 95% CI − 0.91 to − 0.42, *P* < 0.00001), and waking time after persistent sleep onset (WASO, mixed min and %) (SMD = − 0.42, 95% CI − 0.81, − 0.03, *P* = 0.04), with no obvious effect on non-rapid eye movement stage 2 (N2, mixed min and %) (SMD = − 0.15, 95% CI − 0.41 to 0.11, *P* = 0.25), rapid eye movement (REM, mixed min and %) (SMD = 0.22, 95% CI − 0.26 to 0.70, *P* = 0.37), rapid eye movement latency (REML, min) (MD = 2.33, 95% CI − 27.56 to 32.22, *P* = 0.88), or apnea–hypopnea index (AHI) (MD = − 4.21, 95% CI − 14.02 to 5.59, *P* = 0.40). Daytime drowsiness (OR = 2.53, 95% CI 1.14–5.64, *P* = 0.02) and decreased appetite (OR = 2.81, 95% CI 1.14–6.92, *P* = 0.02) occurred with greater frequency in the trazodone group as compared to the control group, and the differences were significant. The results of quality of evidence were very low in TST, N3 and AHI, were low in LPS, WASO and REM, and were moderate in N1 and NAs. The sources of heterogeneity in TST and N3 were not found out from sensitive and subgroup analysis and there was no high quality of evidence in outcomes by GRADE Assessment. Trials with combination of other therapy could be a problem in this meta-analysis as the possibility of interactions were found from sungroup analysis. Trazodone could improve sleep by changing the sleep architecture in insomnia disorder, but it should be used with caution due to the adverse events that may occur.

*PROSPERO registration* register name: The effect of trazodone on polysomnography sleep architecture in patients with insomnia: a systematic review and meta-analysis protocol; Registration Number CRD42020215332.

## Introduction

Insomnia disorder is a prevalent disorder, and approximately one-third of the general population presents insomnia symptoms^1^. Pharmacotherapy is one of the major approaches to the treatment of insomnia disorder^2^. Among medications, trazodone is controversial for its use as a sleeping aid. It is a second-generation triazolopyridine derivative in the category of serotonin antagonist and reuptake inhibitors (SARI) drugs and is used as an antidepressant^3^. However, since the end of the last century, a few studies determined that it possesses sedative and hypnotic action from the antagonistic mechanism at the α1- and α2-adrenergic receptors, 5-HT2A receptors, and histamine H1 receptors^4–6^.

Many clinical trials^7–11^ and reviews^12^ or meta-analyses^13^ have reported trazodone’s general safety and subjective efficacy when used for primary and secondary insomnia. Furthermore, trazodone has been reported to be among the most widely prescribed sleep aids in the United States^12^ and in Nova Scotia, Canada^14^. However, trazodone is not approved by U.S. Food and Drug Administration (FDA) for sleep disorders^3^, and the American Academy of Sleep Medicine Clinical Practice Guideline^2^ did not suggest that clinicians used trazodone as a treatment for sleep onset or sleep maintenance in adults because the evidence for its use for those purposes was limited.

In recent years, with the wide use of polysomnography (PSG), additional studies^15–18^ on the objective efficacy of trazodone in the treatment of insomnia disorder have been reported; thus, it is now possible to understand the effect of trazodone on the sleep architecture of insomnia disorder. A retrospective study^19^ revealed that antidepressants reduced waking time after persistent sleep onset (WASO), prolonged non-rapid eye movement stage 2 (N2) and shortened rapid eye movement (REM) sleep. It provided an indirect evidence for clinicians to use trazodone. Therefore, this systematic review and meta-analysis were designed to evaluate the objective effects of trazodone on insomnia disorder, and especially to assess the changes to sleep architecture based on PSG parameters, to provide additional evidence so that trazodone can be used as a sleeping aid.

## Methods

According to the Preferred Reporting Items for Systematic Reviews and Meta-Analyses protocols (PRISMA-P) 2015 statement^20^, the protocol was previously registered on November 29, 2020. The PROSPERO Registration Number is CRD42020215332. The manuscript was prepared according to PRISMA2020 statement^21^, and a checklist was provided in Supplementary Information 1.

### Inclusion criteria

P: Primary or secondary insomnia, or insomnia comorbid with other disorders. Trials that used either predefined diagnostic criteria or diagnosed patients according to a chief complaint of insomnia in participants were also included.

I: The intervention applied in the experimental group was trazodone alone or in combination with other therapies. The treatment course of trazodone was at least 1 week. The doses of trazodone were not limited.

C: Comparison was placebo or blank, and other concomitant treatments, if any, were consistent between the two groups.

O: Primary outcomes were PSG sleep parameters, including total sleep time (TST), sleep efficiency (SE), latency to onset of persistent sleep (LPS), waking time after persistent sleep onset (WASO), the number of awakenings (NAs) or arousal index (ArI), non-rapid eye movement stage 1 (N1), non-rapid eye movement stage 2 (N2), non-rapid eye movement stage 3 (N3), rapid eye movement (REM), apnea–hypopnea index (AHI), and rapid eye movement latency (REML). Time points were 1 week and above. For some earlier studies, N3 sleep was divided into N3 and non-rapid eye movement stage 4 (N4). However, according to the American Academy of Sleep Medicine manual for the scoring of sleep and associated events (2007), N4 was no longer separated from the other non-rapid eye movement sleep (NREM) sleep stages^22^. In this study, we only considered N3. The secondary outcomes were adverse events and discontinuation for all causes. According to actual data, amendments to the registration protocol consisted of adding REML to outcomes and mean arterial oxygen saturation (SaO2), the periodic limb movements (PLM) index, and N4 were removed.

### Other eligibility criteria

Articles describing randomized controlled trials (RCTs) published in either English or Chinese were included in the analysis.

### Exclusion criteria

The following studies were excluded: (1) duplicate or those with overlapping populations; (2) quasi-random; (3) those with unavailable data after attempting to contact the authors; and (4) those without PSG parameters in the outcomes.

### Search and screen strategy

PubMed, EMBASE, Web of Science, PsycINFO, Cochrane Library, Chinese Biomedical Literature Database (SinoMed), China National Knowledge Infrastructure, Wanfang Database, and the China Science and Technology Journal Database were searched from inception to June 2022 employing the following keywords: “sleep initiation and maintenance disorders,” “insomnia,” and “trazodone.” T.L. and J.W. independently searched the above databases. Inconsistencies were discussed among them, or they consulted with the third author, Y.Z. According to the different retrieval modes, keywords were combined with free words to perform a comprehensive search strategy  (Supplementary Information 2). After excluding duplicate studies, T.L. and J.W. independently screened the titles and abstracts. Then, full-text articles were assessed for eligibility according to the inclusion criteria, and reasons for exclusion were noted. If disagreements existed, a consensus would be reached through discussion among our team.

### Data extraction and quality assessment

T.L. and J.W. independently extracted data from the included studies. Any disagreement was resolved by discussion until consensus was reached or by consulting with Y.Z. The data were recorded in an Excel table format and included categories of the first author, publication year, study country, sex, sample size, age, diagnosis, intervention, trazodone doses and duration, PSG parameter outcomes, discontinuation for all causes, and adverse and discontinuation events. For the crossover design study, only phase 1 data were extracted if two phases were separately reported and the washout period was not reported in the trial design. If any data were missing, we would calculate it from existing data or contact the author(s) to obtain the missing information.

The risk of bias from the included studies was assessed by guidance from the Cochrane Handbook for Systematic Reviews of Interventions^23^. T.L. and J.W. used Revised tool for Risk of Bias in randomized trials (RoB 2)^24^ to independently score the included studies as low, some concerns, or high risk in five domains. When inconsistencies arose, they were resolved by consensus through discussion by the team.

### Data analysis

Cochrane Review Manager v5.3 was used to process the data. Mean differences (MD, when the units of data were unified) or standardized mean differences (SMD, when the units of data were not uniform), as well as 95% confidence intervals (CIs), were estimated as continuous measures. The odds ratios (OR), as well as the 95% CIs, were estimated as dichotomous measures (the number of discontinued patients). When standard deviation (SD) were missing and could not be obtained from the authors, they were calculated from reported *P* values, *t* values, confidence intervals (CIs), or standard errors^25^. Trials reporting median results were excluded.

Heterogeneity was evaluated, and *I*^2^ ≥ 75%, *I*^2^ ≥ 50%, and *I*^2^ ≥ 25% represented large, moderate, and small heterogeneity, respectively^26^. When *I*^2^ < 50%, the fixed effects model was used. When *I*^2^ ≥ 50%, the random effects model was used, and then sensitivity and subgroup analyses were performed to determine possible sources of the heterogeneity and to assess robustness of the synthesized results depending on the available data^23^. The results of the meta-analysis are presented as forest plots and tables. Publication biases were assessed by funnel plots and Egger’s tests^23,27^ (Stata 15.1, Stata Corp., College Station, TX, USA). Two-sided *P* < 0.05 was considered statistically significant. The results of outcomes were assessed the quality of evidence by the Grading of Recommendations Assessment, Development and Evaluation (GRADE) under the software GRADE profiler (https://gradeprofiler.software.informer.com/download/).

## Results

### Characteristics of included studies

**Figure 1:**
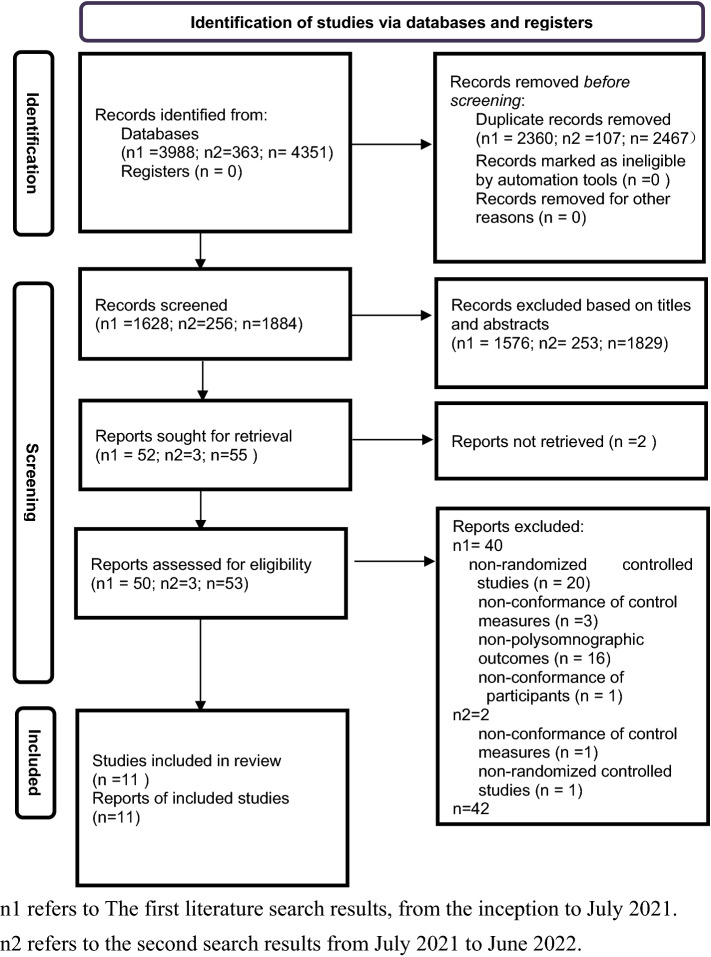
Flow diagram of the study selection process. n1 refers to The first literature search results, from the inception to July 2021. n2 refers to the second search results from July 2021 to June 2022.

Eleven RCTs^9,15–18,28–33^ published between 1999 and 2021 and involving 466 participants were selected. The procedure used for study selection is shown in the flow diagram in Revised Fig. 1. Among included trials, four^9,29–31^ trials (36.4%) recruited patients from European countries, five^16–18,28,33^ trials (45.4%) recruited from Asia, and two^15,32^ (18.2%) trials recruited from North America. Three trials^29,31,32^ were crossover RCTs. Eight^9,15,28–33^ trials involved patients with mean age below 60 years, and three^16–18^ trials enrolled patients older than 60 years of age. Two^9,32^ trials involved primary insomnia, four^16–18,28^ trials involved comorbid internal disorders, and five^15,29–31,33^ trials involved comorbid psychiatric disorders. Nine^9,15,17,28–33^ trials employed diagnosis by International standard criteria, one^16^ trials by predefined criteria, and one^18^ trials by insomnia symptoms. A blank control was selected for three studies^9,16,28^, and a placebo control was selected for eight studies^15,17,18,29–33^.

The daily dose of trazodone ranged from 25 to 400 mg. Among ten trials, three^16,30,33^ trials reported progressive doses (50–400 mg per day), and eight^9,15,17,18,28,29,31,32^ trials reported a fixed dose of 100 mg, 50 mg, or 25 mg per day. The course of treatment ranged from 1 week to 3 months, with four trials^18,29,31,32^ being reported for 1 week and six^9,15–17,30,33^ trials for 1 month or longer. Seven trials^9,15,17,29,31–33^ referred to the management of the ‘first night’ effect during polysomnography, while the other four^16,18,28,30^ trials did not refer to it. Two^18,30^trials reported data in both the median and mean, nine^9,15–17,28,29,31–33^ trials only reported the mean ± standard deviation, and standard deviations were absent from two studies’ data^15,30^. Six^15,17,18,29–31^ trials reported sleep stages as percentages, and four studies^9,16,32,33^ trials reported them in minutes. Table 1 summarizes these characteristics.

**Table 1:** Summary of the characteristics of the included studies.

Study	Sample size (experimental/control)	Sex (M/F)	Age (mean ± SD)	Diagnosis	Intervention		Dosage	Duration	Discontinuation (experimental/control)	PSG outcomes	Adverse events
					Experimental	Control					
Cao et al.^16^	(36/37)	41/32	61.33 ± 2.82	Stroke and abnormal PSQI	Flupentixol-Melitracen and trazodone	Flupentixol-Melitracen	100–400 mg/d	1 month	0/0	TST, N3, REM	Not mentioned
Zhang et al.^33^	(20/18)	Not mentioned	45.7 ± 9.5	Insomnia with benzodiazepine-dependence	Trazodone	Placebo	50–300 mg/d	3 months	0/2	TST, SE, LPS, N3	No adverse effects were found
Roth et al.^32^	(16/16)	(8/24)	44 ± 11	Primary insomnia	Trazodone	Placebo	25 mg/d	1 week	0/0	SE, LPS, NAs, WASO, REM, N1, N2, N3, REML	Impaired next-day memory performance, equilibrium, and muscle endurance
Le Bon et al.^30^	(8/8)	(15/1)	43.8 ± 8.3	Insomnia with alcohol post-withdrawal syndrome	Trazodone	Placebo	50–200 mg/d	1 month	1/1	TST, NAs, WASO, N3, REM, AHI, REML, NAs, ArI	Hangover, dizziness, headaches, and skin irritation
Haffmans and Vos^29^	(3/4)	Not mentioned	44	Sleep disorder induced by brofaromine	Trazodone	Placebo	50 mg/d	1 week	0/0	TST, LPS, SE, NAs, REML, N1, N2, N3, REM	Not mentioned
Kaynak et al.^31^	(12/12)	(0/24)	42 ± 9	Major depression comorbid with insomnia disorder	Trazodone	Placebo	100 mg/d	1 week	0/0	TST, LPS, SE, NAs, REML, N1, N2, N3, REM	Mild and transient acid indigestion, mild daytime sedation in the morning
Wang et al.^17^	(16/14)	(15/15)	62.87 ± 11.94	Arteriosclerotic cerebral small vessel disease comorbid with chronic insomnia	Trazodone	Placebo	50 mg/d	1 month	4/6	TST, LPS, SE, WASO, N1, N2, N3, REM, AHI, ArI	Insomnia deterioration, akathisia, nausea, loss of appetite, dizziness, and headache
Stein et al.^15^	(63/56)	Not mentioned	38.2 ± 8.6	Sleep disturbance during methadone maintenance	Trazodone	Placebo	50 mg/d	1 month	5/7	TST, SE, WASO, N1, N2, N3, REM, AHI, ArI	Increased thirst or dry mouth and decreased appetite
Chen et al.^18^	(22/22)	(24/20)	61.7 ± 10.6	Obstructive sleep apnea after ischemic stroke and insomnia symptoms	Trazodone	Placebo	100 mg/d	1 week	2/2	SE, N3, REM, AHI, ArI	No obvious adverse effects
Zavesicka lucie et al.^9^	(10/10)	(5/15)	47.4 ± 12.6	Primary insomnia	CBTI and trazodone	CBTI	100 mg/d	2 months	0/0	TST, LPS, SE, WASO, N1, N2, N3, REM	Not mentioned
Li et a.l^28^	(31/32)	(29/34)	55.31 ± 7.45	Chronic insomnia comorbid type 2 diabetes	rTMS and trazodone	rTMS	50 mg/d	2w	5/4	TST, LPS, NAs	Dizziness, headaches

*AHI* apnea–hypopnea index, *ArI* arousal index, *CBTI* cognitive behavior therapy for insomnia, *F* female, *LPS* latency to onset of persistent sleep, *M* male, *N1* non-rapid eye movement stage 1, *N2* non-rapid eye movement stage 2, *N3* non-rapid eye movement stage 3, *NAs* the number of awakenings, *PSG* polysomnography, *PSQI* Pittsburgh Sleep Quality Index, *REM* rapid eye movement, *REML* rapid eye movement latency, *SD* standard deviation, *SE* sleep efficiency, *TST* total sleep time, *WASO* wakefulness after persistent sleep onset.

### Outcomes

Forests plots and funnel plots are given in  Supplementary Information 3, and the results are summarized in tables.

### Primary outcomes

The results were summarized and appear in Table 2. Compared with the control group, trazodone significantly increased TST (MD = 39.88, 95% CI 14.44–65.32, *P* = 0.002) and N3 (SMD = 1.61, 95% CI 0.69–2.53, *P* = 0.0006); trazodone significantly decreased LPS (MD = − 19.30, 95% CI − 37.28 to − 1.32, *P* = 0.04), N1 (SMD = − 0.62, 95% CI − 1.13 to − 0.12, *P* = 0.02), NAs (SMD = − 0.67, 95% CI − 0.91 to − 0.42, *P* < 0.00001), and WASO (SMD = − 0.42, 95% CI − 0.81, − 0.03, *P* = 0.04). There was no obvious effect of trazodone on N2 (SMD = − 0.15, 95% CI − 0.41 to 0.11, *P* = 0.25), REM (SMD = 0.22, 95% CI − 0.26 to 0.70, *P* = 0.37), SE (MD = 7.94, 95% CI − 8.92 to 24.81, *P* = 0.36) or REML (MD = 2.33, 95% CI − 27.56 to 32.22, *P* = 0.88). The sedative effect of trazodone did not worsen AHI (MD = − 4.21, 95% CI − 14.02 to 5.59, *P* = 0.40).

**Table 2:** Summary of pooled effects of trazodone on PSG parameters for insomnia disorder (MDs or SMDs based on differences in values at follow-up).

Outcomes	Heterogeneity	MD/SMD	95% CI	Z	*P*	Egger’s test (P)
TST^9,15–17,28,29,31,33^	*P* < 0.00001; *I*^2^ = 82%	39.88	14.44, 65.32	3.07	0.002	0.328
SE (%)^9,15,17,32,33^	*P* < 0.00001; *I*^2^ = 98%	7.94	− 8.92, 24.81	0.92	0.36	0.144
LPS^9,17,28,29,31–33^	*P* < 0.00001; *I*^2^ = 97%	− 19.30	− 37.28, − 1.32	2.10	0.04	0.085
N1^9,15,17,29,31,32^	*P* = 0.02; *I*^2^ = 62%	− 0.62^a^	− 1.13, − 0.12	2.42	0.02	0.066
N2^9,15,17,29,31,32^	*P* = 0.28; *I*^2^ = 20%	− 0.15^a^	− 0.41, 0.11	1.15	0.25	0.185
N3^9,15–18,29,31–33^	*P* < 0.00001; *I*^2^ = 93%	1.61^a^	0.69, 2.53	3.43	0.0006	0.043
REM^9,15–17,29,31,32^	*P* = 0.001; *I*^2^ = 73%	0.22^a^	− 0.26, 0.70	0.90	0.37	0.622
REML^29,31,32^	*P* = 0.54; *I*^2^ = 0%	2.33	− 27.56, 32.22	0.15	0.88	0.198
NAs^15,17,28,29,31,32^	*P* = 0.11; *I*^2^ = 44%	− 0.67^a^	− 0.91, − 0.42	5.31	< 0.00001	0.357
WASO^9,17,29,30,32^	*P* = 0.49; *I*^2^ = 0%	− 0.42^a^	− 0.81, − 0.03	2.09	0.04	0.609
AHI^15,17,18^	*P* = 0.10; *I*^2^ = 57%	− 4.21	− 14.02, 5.59	0.84	0.40	0.558

*AHI* apnea–hypopnea index, *CI* confidence interval, *LPS* latency to onset of persistent sleep, *MD* mean differences, *N1* non-rapid eye movement stage 1, *N2* non-rapid eye movement stage 2, *N3* non-rapid eye movement stage 3, *NAs* the number of awakenings, *PSG* polysomnography, *REM* rapid eye movement, *REML* rapid eye movement latency, *SE* sleep efficiency, *SMD* standardized mean differences, *TST* total sleep time, *WASO* wakefulness after persistent sleep onset.

^a^SMD.

### Sensitive analysis

No obvious heterogeneities were found in N2, REML, NAs, and WASO. Sensitivity analysis of the remaining PSG parameters indicated that the Zhang trial (2013)^33^ was the main heterogeneity source of SE (%), and LPS, the Stein trial (2012)^15^ was the main source of N1, and the Lanfang Cao trial (2018)^16^ was the main source of REM. After elimination of related trial, heterogeneity decreased and the synthesized results were SE(*I*^2^ = 0%, MD = 1.32, 95% CI − 2.06 to 4.70, *P* = 0.44), LPS(*I*^2^ = 10%, MD = − 9.85, 95% CI − 15.34 to − 4.37, *P* = 0.0004), N1(*I*^2^ = 2%, SMD = − 0.86, 95% CI − 1.25 to − 0.46, *P* < 0.0001), and REM(*I*^2^ = 0%, SMD = 0.04, 95% CI − 0.21 to 0.30, *P* = 0.73). Robustness of the synthesized results was well kept. The sensitivity analysis did not determine the main heterogeneity source of TST and N3. The heterogeneity of AHI was from the only trial that enrolled participants with comorbid obstructive sleep apnea (OSA)^18^. Considering the few studies that were included, a random effects model was directly applied to combine the effect size.

### Subgroup analysis

The subgroups were divided according to the average age, the dosage, the course of medication, and the combination of therapies.

According to the average age of participants, subgroups were divided into mean age ≥ 60 years and < 60 years. Trazodone was effective in both subgroups and there was no statistically significant difference between the subgroups for the outcomes of TST (*P*^2^ = 0.81) and N3 (*P*^2^ = 0.72) (Table 3). Difference of age might not be the heterogeneity source of TST and N3.

**Table 3:** Subgroups based on the mean age of participants.

Outcomes	Heterogeneity (*I*^2^) (%)	MD/SMD	95% CI	Z	*P*^1^	*P*^2^
**Mean age ≥ 60 years**						
TST^16,17^	0	42.74	23.05, 62.42	4.26	< 0.0001	0.81
N3^16–18^	86	1.49^a^	0.45, 2.53	2.80	0.005	0.72
**Mean age < 60 years**						
TST^9,15,28,29,31^	85	37.97	4.68, 71.25	2.24	0.03	
N3^9,15,29,31–33^	93	1.61^a^	0.69, 2.53	2.65	0.008	

*CI* confidence interval, *MD* mean differences, *N3* non-rapid eye movement stage 3, *P*^*1*^ variation within the group, *P*^*2*^ differences between subgroups, *SMD* standardized mean differences, *TST* total sleep time.

^a^SMD.

According to the maximum daily dose of the drug, two subgroups were created, consisting of trazodone at ≥ 100 mg/day and ≤ 50 mg/day. High-dose trazodone was more effective than the control for decreasing N2 (SMD = − 0.83, 95% CI − 1.45 to − 0.21, *P* = 0.009) and increasing N3 (SMD = 2.80, 95% CI 1.31–4.28, *P* = 0.0002), and the subgroup differences were significance (*P*^2^ = 0.02; *P*^2^ = 0.002). The high dose might be more optimal for increasing TST (MD = 49.48, 95% CI 13.24–85.72, *P* = 0.007), but there was no statistical difference between subgroups (*P*^*2*^ = 0.40). The high dose may be more effective for decreasing LPS (MD: = − 39.36, 95% CI − 40.64 to − 38.09, *P* < 0.00001), and showed statistical difference between subgroups (*P*^2^ < 0.00001). The high dose was effective for decreasing N1 (SMD = − 1.04, 95% CI − 1.96 to − 0.11, *P* = 0.03), and there was no significant difference (*P*^*2*^ = 0.28) between the dose-subgroups. As for REM sleep, the dose level had no effect on it. Difference of dosage could be the heterogeneity source of LPS and N2. Due to the limited samples of the included literature, it is impossible to distinguish the difference between the gradually increasing dosage model and the fixed low-dose model (Table 4).

**Table 4:** Subgroups based on the different dosages of trazodone.

Outcomes	Heterogeneity (*I*^2^) (%)	MD/SMD	95% CI	Z	*P*^1^	*P*^2^
**Dosage ≥ 100 mg/d**						
TST^9,16,31,33^	86	49.48	13.24, 85.72	2.68	0.007	0.40
LPS^9,31,33^	0	− 39.36	− 40.64, − 38.09	60.57	< 0.00001	< 0.00001
N1^17,29^	51	− 1.04^a^	− 1.96, − 0.11	2.19	0.03	0.28
N2^9,31^	0	− 0.83^a^	− 1.45, − 0.21	2.61	0.009	0.02
N3^9,16,18,31,33^	93	2.80^a^	1.31, 4.28	3.69	0.0002	0.002
REM^9,31^	86	0.39^a^	− 0.73, 1.50	0.68	0.49	0.60
**Dosage ≤ 50 mg/d**						
TST^15,17,28,29^	73	25.62	− 15.90, 67.14	1.21	0.23	
LPS^17,28,29,32^	4	− 9.02	− 12.88, − 5.16	4.58	< 0.00001	
N1^9,15,31,32^	56	− 0.44^a^	− 0.98, 0.10	1.59	0.11	
N2^15,17,29,32^	0	− 0.01^a^	− 0.30, 0.28	0.06	0.95	
N3^15,17,29,32^	34	0.33^a^	− 0.09, 0.75	1.52	0.13	
REM^15,17,29,32^	0	0.08^a^	− 0.20, 0.36	0.57	0.57	

*CI* confidence interval, *LPS* latency to onset of persistent sleep, *MD* mean differences, *N1* non-rapid eye movement stage 1, *N2* non-rapid eye movement stage 2, *N3* non-rapid eye movement stage 3, *P*^*1*^ variation within the group, *P*^*2*^ differences between subgroups, *REM* rapid eye movement, *SMD* standardized mean differences, *TST* total sleep time.

^a^SMD.

According to the course of medication, subgroups were created consisting of a long-treatment group (≥ 1 month) and short-treatment group (1–2 weeks). Long-treatment increased TST (MD = 39.75, 95% CI 4.34–75.17, *P* = 0.03), but the differences between subgroups were not significant (*P*^2^ = 0.88). Short and long courses were effective in decreasing LPS (MD = − 8.47, 95% CI − 12.49 to − 4.45, *P* < 0.0001; MD = − 17.60, 95% CI − 29.83 to − 5.36, *P* = 0.005) and increasing N3 (SMD = 1.27, 95% CI 0.39–2.16, *P* = 0.005; SMD = 2.57, 95% CI 0.88–4.26, *P* = 0.003), and no significant differences in LPS (*P*^2^ = 0.16) or N3 (*P*^2^ = 0.18) were found between subgroups. Short-treatment decreased N1 (SMD = − 1.04, 95% CI − 1.58 to − 0.50, *P* = 0.0002), and the differences between subgroups was significant (*P*^2^ = 0.009). The length of the treatment course had no effect on N2 or REM. The difference of course could be the heterogenity source of N1 (Table 5).

**Table 5:** Subgroups based on different treatment durations.

Outcomes	Heterogeneity (*I*^2^) (%)	MD/SMD	95% CI	Z	*P*^1^	*P*^2^
**Treatment duration (1 week)**						
TST^28,29,31^	72	35.24	− 14.00, 84.47	1.40	0.16	0.88
LPS^28,29,31,32^	0	− 8.47	− 12.49, − 4.45	4.13	< 0.0001	0.16
N1^29,31,32^	35	− 1.04^a^	− 1.58, − 0.50	3.77	0.0002	0.009
N2^29,31,32^	6	− 0.21^a^	− 0.72, 0.29	0.84	0.40	0.93
N3^18,29,31–33^	80	1.27^a^	0.39, 2.16	2.82	0.005	0.18
REM^29,31,32^	0	− 0.03^a^	− 0.48, 0.42	0.13	0.90	0.43
**Treatment duration (≥ 1 month)**						
TST^9,15–17,33^	87	39.75	4.34, 75.17	2.20	0.03	
LPS^9,17^	28	− 17.60	− 29.83, − 5.36	2.82	0.005	
N1^9,15,17^	37	− 0.21^a^	− 0.52, 0.09	1.37	0.17	
N2^9,15,17^	51	− 0.25^a^	− 0.78, 0.27	0.94	0.35	
N3^9,15–17,33^	96	2.57^a^	0.88, 4.26	2.99	0.003	
REM^9,15–17^	84	0.32^a^	− 0.41, 1.04	0.86	0.39	

*CI* confidence interval, *LPS* latency to onset of persistent sleep, *MD* mean differences, *N1* non-rapid eye movement stage 1, *N2* non-rapid eye movement stage 2, *N3* non-rapid eye movement stage 3, *P*^*1*^ variation within the group, *P*^*2*^ differences between subgroups, *REM* rapid eye movement, *SMD* standardized mean differences, *TST* total sleep time.

^a^SMD.

### Analysis on combination with other therapies

Three studies^9,16,28^ used trazodone in combination with other therapies. Leave-one-out were made in N1, N2, WASO and NAs, and the elimination of one relative trial (with a combination therapy) could not change the final results. Subgroup analysis were made in TST, LPS, REM and N3 between combination therapy subgroup and non-combination. Trazodone lost the effect to TST in non-combination group compared with the combination group, but the differences between subgroups were not significant (*P* = *0.69*). Trazodone lost the effect to LPS in both group and the subgroup difference was not significant (*P* = *0.97*) either. Further, the subgroup analysis did not find any change of the results of REM and N3. Combination with other therapy was not found to be a source of heterogeneity from above analysis (Table 6).

**Table 6:** Analysis on combination with other therapies.

Outcomes	Combination therapy			Non-combination therapy			*P*^2^
	*I*^2^ (%)	MD/SMD	*P*^*1*^	*I*^2^ (%)	MD/SMD	*P*^*1*^	
**Sensitivity analysis (Leave-one-out)**							
N1^9,15,17,29,31,32^	62	− 0.62^a^	0.02	69	− 0.40^a^	0.005	
N2^9,15,17,29,31,32^	20	− 0.15^a^	0.25	0	− 0.08^a^	0.56	
WASO^9,17,29,30,32^	0	− 0.42^a^	0.04	0	− 0.52^a^	0.02	
NAs^15,17,28,29,31,32^	44	− 0.67^a^	< 0.00001	0	− 0.52^a^	0.0002	
**Subgroups analysis**							
TST^9,15–17,28,29,31,33^	68	44.08	0.002	85	33.36	0.14	0.69
LPS^9,17,28,29,31–33^	52	− 19.98	0.29	90	− 19.27	0.06	0.97
REM^9,15–17,29,31,32^	91	0.49^a^	0.59	0	0.09^a^	0.52	0.66
N3^9,15–18,29,31–33^	50	2.17^a^	< 0.00001	92	1.43^a^	0.005	0.27

*MD* mean differences, *SMD* standardized mean differences, *TST* total sleep time, *LPS* latency to onset of persistent sleep, *N1* non-rapid eye movement stage 1, *N2* non-rapid eye movement stage 2, *N3* non-rapid eye movement stage 3, *REM* rapid eye movement, *NAs* the number of awakenings, *WASO* wakefulness after persistent sleep onset, *P*^*1*^ variation within the group, *P*^*2*^ differences between subgroups.

^a^ SMD.

### Secondary outcomes

For adverse events, 5 studies^15,17,28,30,31^ reported both mild to moderate discomfort symptoms and the number of cases, but only one^17^ study reported that two participants withdrew due to discomfort after trazodone intake. The most common adverse events reported were daytime drowsiness, dizziness, headache, and decreased appetite. Daytime drowsiness (OR = 2.53, 95% CI 1.14–5.64, *P* = 0.02) and decreased appetite (OR = 2.81, 95% CI 1.14–6.92, *P* = 0.02) occurred more in the trazodone group than that which occurred in the control group, and the differences were significant. Additionally, there were no significant differences for headache (OR = 1.01, 95% CI 0.43–2.36, *P* = 0.99) or dizziness (OR = 2.10, 95% CI 1.00–4.41, *P* = 0.05).All eleven studies reported discontinuations for all causes. There was no significant difference between the trazodone and control groups in discontinuation for all causes (OR = 0.63, 95% CI 0.33–1.18, *P* = 0.15).

Funnel plots revealed asymmetry in TST, LPS, SE (%), N1 and N3 which suggest high posibility of publication bias. And then Publication bias tests were performed for every outcome by egger’s test (Table 2), and significant statistical difference was only found in N3 (*P* = 0.043). However, the accuracy of publication bias test was questionable as the number of included trials in every outcome analysis did not exceed ten^34^.

### Quality assessment to risk of bias

The risk of bias was assessed by the Revised Cochrane risk-of-bias tool for randomized trials (RoB 2) for TST, LPS, NAs, WASO, N1, N3, REM and AHI. All eleven trials were included in. The overall risk of bias to TST was 3 low risks^15,29,31^ and 5 some concerns^9,16,17,28,33^, and overall to LPS was 3 low risks^29,31,32^ and 4 some concerns^9,17,28,33^ and overall to WASO was 2 low risks^29,32^ and 3 some concerns^9,17,30^, and overall to NAs was 4 low risks^15,29,31,32^ and 2 some concerns^17,28^, and overall to AHI was 2 low risks^15,18^ and 1 some concerns^17^, and overall to N1 was 4 low risks^15,29,31,32^ and 2 some concerns^9,17^, and overall to N3 was 5 low risks^15,18,29,31,32^ and 4 some concerns^9,16,17,33^, and overall to REM was 4 low risks^15,29,31,32^ and 3 some concerns^9,16,17^. None of outcomes was found high risk in overall assessment. The overview of quality assessment was given in  Supplementary Information 4.

### Quality of evidence assessment by GRADE

The results of TST, LPS, NAs, WASO, AHI, N1, N3 and REM were assessed the quality of evidence by GRADE. The results qualities of TST, N3 and AHI were very low, and LPS, WASO and REM were low, and NAs and N1 were moderate. None of high quality evidence was found in above outcomes (Table 7).

**Table 7:** Study quality of evidence according to GRADE guideline.

Outcomes	No of participants (studies)	Risk with trazodone	Domain					Certainty of the evidence (GRADE)
			Risk of bias	Inconsistency	Indirectness	Imprecision	Publication bias	
TST^9,15–17,28,29,31,33^	374 (8 studies)	MD 39.88 higher (14.44 to 65.32 higher)	Serious^a^	Very serious^b^	Not serious	Serious^c^	None	Very low
LPS^9,17,28,29,31–33^	214 (7 studies)	MD 19.30 lower (37.28 to 1.32 lower)	Serious^a^	Not serious^d^	Not serious	Serious^c^	None	Low
WASO^9,17,29,30,32^	105 (5 studies)	SMD 0.42 lower (0.81 to 0.03 lower)	Serious^a^	Not serious	Not serious	Serious^c^	None	Low
AHI^15,17,18^	193 (3 studies)	MD 4.21 lower (14.02 lower to 5.59 higher)	Not serious	Serious^e^	Not serious	Very serious^f^	None	Very low
N1^9,15,17,29,31,32^	232 (6 studies)	SMD 0.62 lower (1.13 to 0.12 lower)	Not serious	Not serious^g^	Not serious	Serious^c^	None	Moderate
N3^9,15–18,29,31–33^	387 (9 studies)	SMD 1.61 higher (0.69 to 2.53 higher)	Not serious	Very serious^h^	Not serious	Serious^c^	None	Very low
REM^9,15–17,29,31,32^	326 (7 studies)	SMD 0.22 higher (0.26 lower to 0.7 higher)	Not serious	Not serious^i^	Not serious	Very serious^f^	None	Low
NAs^15,17,28,29,31,32^	275 (6 studies)	SMD 0.67 lower (0.91 to 0.42 lower)	Not serious	Not serious	Not serious	Serious^c^	None	Moderate

*GRADE* Grading of recommendations assessment, development and evaluation, *MD* Mean difference, *SMD* standardized mean difference, *TST* Total sleep time, *LPS* latency to onset of persistent sleep, *WASO* wakefulness after persistent sleep onset, *AHI* Apnea–hypopnea index, *N1* non-rapid eye movement stage 1, *N3* non-rapid eye movement stage 3, *NAs* the number of awakenings, *REM* Rapid eye movement sleep.

^a^According to Revised Cochrane risk-of-bias tool for randomized trials (RoB 2), most studies included in this meta-analysis were evaluated to have significant risk of bias concerns.

^b^Significant unexplained heterogeneity (I^2^ = 82%).

^c^Serious imprecision due to the small sample size (< 400 participants).

^d^Significant heterogeneity (I^2^ = 97%) can be explained by sensitive and subgroup analysis.

^e^Moderate unexplained heterogeneity (I^2^ = 57%).

^f^Very serious imprecision due to the small sample size (< 400 participants) and wide confidence interval.

^g^Moderate heterogeneity (I^2^ = 62%) can be explained by sensitivity analysis.

^h^Significant unexplained heterogeneity (I^2^ = 93%).

^i^Moderate heterogeneity (I^2^ = 73%) can be explained by sensitivity analysis.

## Discussion

The present meta-analysis focused on the effect of trazodone on sleep architecture and found that trazodone could increase TST and reduce LPS, but has no effect on SE. It is mainly because trazodone may play a more important role in optimizing the internal structure of sleep, such as reducing N1 and NAs, and increasing N3. This effect has more advantages than the currently commonly used hypnotics, especially benzodiazepine sleeping aids. At present, it is believed that many hypnotics could reduce N3 and REM sleep, mainly increase N2 sleep. And most of them have the risk of excessive morning sedation and damage cognitive function^35,36^. However, some clinical studies have found that trazodone could improve the sleep of Alzheimer disease without affecting cognitive function^10,37^,and trazodone had a negative effect on the ability to drive vehicles^32^. The mechanism of trazodone improving sleep structure could be associated with blocking 5-HT2 serotonin receptors, H1 histamine receptors and alpha-1 adaptive receptors^38,39^, while some hypnotics were agonists of gamma-aminobutyric acid GABAA receptors^40^. In addition, some studies have found that antidepressants had effect on REM sleep^19,39^, but the opposite conclusion was confirmed in our study. The reason may be that patients with depression have increased REM sleep and shortened REML^41^, and most of the people included in this meta-analysis did not have the problem of comorbid depression. Therefore, the impact on REM and REML was not shown. Finally, although the sample size of AHI included in this study was limited, the current study revealed trazodone had a negative effect on AHI. One latest clinical study also found that trazodone reduces AHI and increases the awakening threshold of obstructive sleep apnea after ischemic stroke^18^. It might be another advantage of Trazodone in clinical application as the higher prevalency of insomnia symptoms in patients with OSA (40–60%) compared to that observed in the general population^42^. Further, it is worth discussing whether trazodone is more suitable for comorbid insomnia and OSA patients to improve their sleep quality or to improve their compliance with continuous positive airway pressure (CPAP) treatment.

Subgroup analyses were conducted according to the stratification of average age, dosage, course of treatment, and whether combined with other therapy. It revealed that the dose of trazodone was the main source of heterogeneity for LPS. When it was more than 100 mg/d, it could better reduce LPS than the fixed low dose of 25-50 mg/d. Therefore, for patients with difficulty in sleep initiation or patients with depression, a higher dose might be more reasonable^35^. Compared with the treatment course of more than 1 month, the treatment course of 1 week was better to reduce N1 sleep. It was suggested that the effect of trazodone may be reduced in long-term application, which was similar to benzodiazepine receptor agonists^43^. However, it may also be resulted from the trial of stein (2012)^15^ as it was found to be the source of N1’s heterogeneity, and once leave it out, the subgroup difference was not significant (*P* = 0.33). In recent years, many studies^19,36^ have revealed the effect of other psychotropic drugs on the PSG results such as antidepressants, anticonvulsants, and antiepileptic drugs. Though it was not discussed enough on the safety, It is believed that such exploration has practical significance for the treatment of comorbid insomnia.

Our meta-analysis has several improvements and advantages compared to previous research. First, the results of a previous meta-analysis^13^ indicated that trazodone did not affect TST, LPS, or WASO. This inconsistency may be because the limitations of the included sample size in the previous meta-analysis were greatly affected by the results of Stein’s study^15^ (accounting for 66% of the total included sample size). as enlarged in many more samples, the positive effects were revealed. Second, because the data were obtained from different types of sleep monitoring equipment, this might also affect the accuracy of the results. Our analysis was based on polysomnographic sleep data and revealed that trazodone impacted sleep architecture. Third, for adverse events, previous study^13^ have reported no significant difference between the trazodone group and the placebo group, but our study revealed daytime drowsiness and decreased appetite were significant so that it should be used with caution for clinicians.

Overall, our meta-analysis indicated that the use of trazodone may be a potentially effective treatment in patients with insomnia disorder. These findings may provide references for accurate selection of patients, and also provide direction for future clinical research in exploring the appropriate population, dose, and treatment course for trazodone. However, limited by small sample sizes and participants, and the low quality of some included studies in our study, a larger sample and high quality RCTs on the treatment of trazodone in patients with insomnia disorder are still needed in the future.

### Limitations

This meta-analysis had some limitations. First, while the heterogeneity of most analyses is explained, we still cannot explain the heterogeneity of a few analyses of outcomes due to the limited sample size. Second, trials with the combination of other therapy could be a problem in this meta-analysis as the possibility of interactions were found from subgroup analysis. Third, due to the limited samples and limited literature included, it was impossible to make more analysis on AHI, of which the recommendation for trazodone to improve insomnia in patients with OSA was very low. The last, the present study was not able to distinguish between the gradually increasing dosage model and the fixed low-dose model as sample limitation.

## Conclusion

Trazodone improved sleep by changing the sleep architecture in insomnia patients, increasing TST and N3 sleep, and decreasing LPS, N1, NAs and WASO, and there was no significant effect on N2, REML, REM, SE, or AHI. The recommendation of evidences were from very low to moderate. However, considering its potential adverse events such as daytime drowsiness and loss of appetite, trazodone should be used with caution.

## Supplementary Information

Supplementary Information 1.

Supplementary Information 2.

Supplementary Information 3.

Supplementary Information 4.

## Data Availability

All data generated or analysed during this study are included in this published article and its supplementary information files.

## References

[1] Ohayon MM (2002). Epidemiology of insomnia: What we know and what we still need to learn. Sleep Med. Rev..

[2] Sateia MJ, Buysse DJ, Krystal AD, Neubauer DN, Heald JL (2017). Clinical practice guideline for the pharmacologic treatment of chronic insomnia in adults: An American academy of sleep medicine clinical practice guideline. J. Clin. Sleep Med..

[3] Shin, J. J. & Saadabadi, A. *Trazodone*. https://www.ncbi.nlm.nih.gov/books/NBK470560/ (StatPearls, Updated 2 May 2022).

[4] Tatsumi M, Groshan K, Blakely RD, Richelson E (1997). Pharmacological profile of antidepressants and related compounds at human monoamine transporters. Eur. J. Pharmacol..

[5] Richelson E, Souder T (2000). Binding of antipsychotic drugs to human brain receptors focus on newer generation compounds. Life Sci..

[6] Stahl SM (2009). Mechanism of action of trazodone: A multifunctional drug. CNS Spectr..

[7] Montgomery I, Oswald I, Morgan K, Adam K (1983). Trazodone enhances sleep in subjective quality but not in objective duration. Br. J. Clin. Pharmacol..

[8] Wichniak A (2007). The effectiveness of treatment with trazodone in patients with primary insomnia without and with prior history of hypnotics use. Pol Merkur Lekarski.

[9] Zavesicka L (2008). Trazodone improves the results of cognitive behaviour therapy of primary insomnia in non-depressed patients. Neuro Endocrinol. Lett..

[10] Camargos EF (2014). Trazodone improves sleep parameters in Alzheimer disease patients: A randomized, double-blind, and placebo-controlled study. Am. J. Geriatr. Psychiatry.

[11] Morin CM (2020). Effectiveness of sequential psychological and medication therapies for insomnia disorder: A randomized clinical trial. JAMA Psychiat..

[12] Jaffer KY (2017). Trazodone for Insomnia: A systematic review. Innov Clin. Neurosci..

[13] Yi XY (2018). Trazodone for the treatment of insomnia: A meta-analysis of randomized placebo-controlled trials. Sleep Med..

[14] Neville HL (2020). Point prevalence survey of benzodiazepine and sedative-hypnotic drug use in hospitalized adult patients. Can. J. Hosp. Pharm..

[15] Stein MD (2012). Trazodone for sleep disturbance during methadone maintenance: A double-blind, placebo-controlled trial. Drug Alcohol Depend..

[16] Cao LF, Luo LH, Fan YH (2018). Therapeutic effects of flupentixol and melitracen tablets combined with trazodone on stroke sleep disorder and their effects on bad emotion of patients. Hebei Med. J..

[17] Wang J (2020). Effects of trazodone on sleep quality and cognitive function in arteriosclerotic cerebral small vessel disease comorbid with chronic Insomnia. Front. Psychiatry.

[18] Chen CY, Chen CL, Yu CC (2021). Trazodone improves obstructive sleep apnea after ischemic stroke: A randomized, double-blind, placebo-controlled, crossover pilot study. J. Neurol..

[19] Ghossoub E, Geagea L, Kobeissy F, Talih F (2021). Comparative effects of psychotropic medications on sleep architecture: A retrospective review of diagnostic polysomnography sleep parameters. Sleep Sci..

[20] Moher D (2015). Preferred reporting items for systematic review and meta-analysis protocols (PRISMA-P) 2015 statement. Syst. Rev..

[21] Page MJ (2021). The PRISMA 2020 statement: An updated guideline for reporting systematic reviews. BMJ.

[22] Iber, C., Ancoli-Israel, S., Chesson, A. L. & Quan, S. F. *The AASM Manual for the Scoring of Sleep and Associated Events: Rules, Terminology and Technical Specifications*, 1st ed. Westchester, IL: American Academy of Sleep Medicine (2007).

[23] Higgins, J. P. *et al.* Cochrane Handbook for Systematic Reviews of Interventions version 6.3. *Cochrane*, https://www.training.cochrane.org/handbook. (Updated February 2022).

[24] Sterne JAC (2019). RoB 2: A revised tool for assessing risk of bias in randomised trials. BMJ.

[25] Furukawa TA, Barbui C, Cipriani A, Brambilla P, Watanabe N (2006). Imputing missing standard deviations in meta-analyses can provide accurate results. J. Clin. Epidemiol..

[26] Higgins JP, Thompson SG, Deeks JJ, Altman DG (2003). Measuring inconsistency in meta-analyses. BMJ.

[27] Sterne JA (2011). Recommendations for examining and interpreting funnel plot asymmetry in meta-analyses of randomised controlled trials. BMJ.

[28] Li, X. M. *Repetitive Transcranial Magnetic Stimulation Combined with Trazodone Treatment Diabetes Mellitus Insomniac Related Research.*, Vol. Dissertation for the master degree, (Zhengzhou University, 2021).

[29] Haffmans PM, Vos MS (1999). The effects of trazodone on sleep disturbances induced by brofaromine. Eur. Psychiatry.

[30] Le Bon O (2003). Double-blind, placebo-controlled study of the efficacy of trazodone in alcohol post-withdrawal syndrome: Polysomnographic and clinical evaluations. J. Clin. Psychopharmacol..

[31] Kaynak H, Kaynak D, Gözükirmizi E, Guilleminault C (2004). The effects of trazodone on sleep in patients treated with stimulant antidepressants. Sleep Med..

[32] Roth AJ, McCall WV, Liguori A (2011). Cognitive, psychomotor and polysomnographic effects of trazodone in primary insomniacs. J. Sleep Res..

[33] Zhang HJ, Jiang XF, Ma MM, Zhang JW (2013). A control study on treatment for benzodiazepine dependence with trazodone. Chin. J. Contemp. Neurol. Neurosurg..

[34] Lin L, Chu H (2018). Quantifying publication bias in meta-analysis. Biometrics.

[35] Wichniak A, Wierzbicka AE, Jarema M (2021). Treatment of insomnia: Effect of trazodone and hypnotics on sleep. Psychiatr. Pol..

[36] Yeh WC (2021). The impact of antiseizure medications on polysomnographic parameters: A systematic review and meta-analysis. Sleep Med..

[37] La AL (2019). Long-Term Trazodone Use and cognition: A potential therapeutic role for slow-wave sleep enhancers. J. Alzheimers Dis..

[38] Jarema M (2011). Trazodon–the antidepressant: Mechanism of action and its position in the treatment of depression. Psychiatr. Pol..

[39] Wichniak A, Wierzbicka A, Walęcka M, Jernajczyk W (2017). Effects of antidepressants on sleep. Curr. Psychiatry Rep..

[40] Nutt D (2006). GABAA receptors: Subtypes, regional distribution, and function. J. Clin. Sleep Med..

[41] Wichniak A, Wierzbicka A, Jernajczyk W (2013). Sleep as a biomarker for depression. Int. Rev. Psychiatry.

[42] Ragnoli B, Pochetti P, Raie A, Malerba M (2021). Comorbid insomnia and obstructive sleep apnea (COMISA): Current concepts of patient management. Int. J. Environ. Res. Public Health.

[43] Riemann D, Perlis ML (2009). The treatments of chronic insomnia: A review of benzodiazepine receptor agonists and psychological and behavioral therapies. Sleep Med. Rev..

