# Supplementary material for: Trazodone changed the polysomnographic sleep architecture in insomnia disorder: a systematic review and meta-analysis
Source: Sci Rep. 2022 Aug 24;12:14453. doi: 10.1038/s41598-022-18776-7 (PMC9402537; doi:10.1038/s41598-022-18776-7)
Supplement: Supplementary file 2 — Supplementary Information 2. [file 41598_2022_18776_MOESM2_ESM.pdf]

PUBMED search strategy

No.	Query
#1	"Trazodone"[Mesh]
#2	(((((((((((((((((((((((((((Tradozone[Title/Abstract]) OR (AF-1161[Title/Abstract])) OR (AF 1161[Title/Abstract])) OR (AF1161[Title/Abstract])) OR (Deprax[Title/Abstract])) OR (Deprax[Title/Abstract])) OR (Gen-Trazodone[Title/Abstract])) OR (Gen Trazodone[Title/Abstract])) OR (Molipaxin[Title/Abstract])) OR (Novo-Trazodone[Title/Abstract])) OR (Novo Trazodone[Title/Abstract])) OR (Trittico[Title/Abstract])) OR (PMS-Trazodone[Title/Abstract])) OR (PMS Trazodone[Title/Abstract])) OR (Ratio-Trazodone[Title/Abstract])) OR (Ratio Trazodone[Title/Abstract])) OR (Ratio Trazodone[Title/Abstract])) OR (Thombran[Title/Abstract])) OR (Trazodon Hexal[Title/Abstract])) OR (Trazodon-Neuraxpharm[Title/Abstract])) OR (Trazodon Neuraxpharm[Title/Abstract])) OR (TrazodonNeuraxpharm[Title/Abstract])) OR (Trazodone Hydrochloride[Title/Abstract])) OR (Trazon[Title/Abstract])) OR (Apo-Trazodone[Title/Abstract])) OR (Apo Trazodone[Title/Abstract])) OR (Nu-Trazodone[Title/Abstract])) OR (Nu Trazodone[Title/Abstract])
#3	"Sleep Initiation and Maintenance Disorders"[Mesh]
#4	((((((((((((((((((((((((((Insomnia[Title/Abstract]) OR (Disorders of Initiating and Maintaining Sleep[Title/Abstract])) OR (DIMS [Title/Abstract])) OR (Early Awakening[Title/Abstract])) OR (Awakening, Early[Title/Abstract])) OR (Nonorganic Insomnia[Title/Abstract])) OR (Insomnia, Nonorganic[Title/Abstract])) OR (Primary Insomnia[Title/Abstract])) OR (Insomnia, Primary[Title/Abstract])) OR (Transient Insomnia[Title/Abstract])) OR (Insomnia, Transient[Title/Abstract])) OR (Rebound Insomnia[Title/Abstract])) OR (Insomnia, Rebound[Title/Abstract])) OR (Secondary

	Insomnia[Title/Abstract])) OR (Insomnia, Secondary[Title/Abstract])) OR (Sleep Initiation Dysfunction[Title/Abstract])) OR (Dysfunction, Sleep Initiation[Title/Abstract])) OR (Dysfunctions, Sleep Initiation[Title/Abstract])) OR (Sleep Initiation Dysfunctions[Title/Abstract])) OR (Sleeplessness[Title/Abstract])) OR (Insomnia Disorder[Title/Abstract])) OR (Insomnia Disorders[Title/Abstract])) OR (Insomnias[Title/Abstract])) OR (Chronic Insomnia[Title/Abstract])) OR (Insomnia, Chronic[Title/Abstract])) OR (Psychophysiological Insomnia[Title/Abstract])) OR (Insomnia, Psychophysiological[Title/Abstract])
#5	#1 OR #2
#6	#3 OR #4
#7	#5 AND #6

Article title: Trazodone Changed the Polysomnographic Sleep Architecture in Insomnia: A Systematic Review and Meta-Analysis
journal name: Drugs
author names: Yongliang Zheng[1] • Tian Lv[2] • Jingjing Wu[3] • Yumeng Lyu[1]
Affiliation:
[1] School of Rehabilitation Medicine, Jiangsu Vocational College of Medicine, Yancheng, China
[2] Department of Neurology, Zhuji Hospital Affiliated Shaoxing University, Shaoxing, China
[3] Department of Cardiology, Pudong New Area People’s Hospital, Shanghai, China
The corresponding author and e-mail address:
Yongliang Zheng, 12131@jsmc.edu.cn
