# Supplementary material for: Trazodone changed the polysomnographic sleep architecture in insomnia disorder: a systematic review and meta-analysis
Source: Sci Rep. 2022 Aug 24;12:14453. doi: 10.1038/s41598-022-18776-7 (PMC9402537; doi:10.1038/s41598-022-18776-7)
Supplement: Supplementary file 3 — Supplementary Information 3. [file 41598_2022_18776_MOESM3_ESM.pdf]

Forests plots and funnel plots for all parameters

Fig. 1 pooled effects of trazodone on TST

Study or Subgroup	Experimental Mean	Experimental SD	Experimental Total	Control Mean	Control SD	Control Total	Weight	Mean Difference IV, Random, 95% CI
P.M.J. Haffmans 1999	297	66.2	3	359.3	58.1	4	5.2%	−62.30 [−156.39, 31.79]
Z. Lucie 2008	361.9	47	10	359.1	56.7	10	11.3%	2.80 [−42.85, 48.45]
Stein MD 2012	355.9	118.3	63	344.1	118.3	56	11.9%	11.80 [−30.78, 54.38]
W. Jihui 2020	392.48	64.55	16	361.2	60.4	14	11.5%	31.28 [−13.46, 76.02]
H. Kaynak 2004	428	39	12	383	66	12	11.7%	45.00 [1.63, 88.37]
Lanfang Cao 2018	382.41	45.92	36	336.92	49.61	37	15.7%	45.49 [23.57, 67.41]
Xuemin Li 2021	398.87	32.97	31	333.78	50.42	32	15.9%	65.09 [44.12, 86.06]
Hongju Zhang 2013	410.1	24.81	20	321.06	16.97	18	16.9%	89.04 [75.64, 102.44]
**Total (95% CI)**			**191**			**183**	**100.0%**	**39.88 [14.44, 65.32]**

Heterogeneity: $Tau^2 = 948.06$; $Chi^2 = 38.54$, df = 7 ($P < 0.00001$); $I^2 = 82\%$
Test for overall effect: Z = 3.07 (P = 0.002)

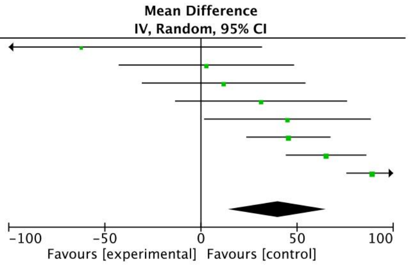

Fig. 2 subgroups analysis to TST (treatment duration)

Study or Subgroup	Experimental Mean	Experimental SD	Experimental Total	Control Mean	Control SD	Control Total	Weight	Mean Difference IV, Random, 95% CI
**2.2.1 1–2W**								
H. Kaynak 2004	428	39	12	383	66	12	11.7%	45.00 [1.63, 88.37]
P.M.J. Haffmans 1999	297	66.2	3	359.3	58.1	4	5.2%	−62.30 [−156.39, 31.79]
Xuemin Li 2021	398.87	32.97	31	333.78	50.42	32	15.9%	65.09 [44.12, 86.06]
**Subtotal (95% CI)**			**46**			**48**	**32.7%**	**35.24 [−14.00, 84.47]**
Heterogeneity: $Tau^2 = 1254.55$; $Chi^2 = 7.04$, df = 2 (P = 0.03); $I^2 = 72\%$								
Test for overall effect: Z = 1.40 (P = 0.16)								
**2.2.2 大于1m**								
Hongju Zhang 2013	410.1	24.81	20	321.06	16.97	18	16.9%	89.04 [75.64, 102.44]
Lanfang Cao 2018	382.41	45.92	36	336.92	49.61	37	15.7%	45.49 [23.57, 67.41]
Stein MD 2012	355.9	118.3	63	344.1	118.3	56	11.9%	11.80 [−30.78, 54.38]
W. Jihui 2020	392.48	64.55	16	361.2	60.4	14	11.5%	31.28 [−13.46, 76.02]
Z. Lucie 2008	361.9	47	10	359.1	56.7	10	11.3%	2.80 [−42.85, 48.45]
**Subtotal (95% CI)**			**145**			**135**	**67.3%**	**39.75 [4.34, 75.17]**
Heterogeneity: $Tau^2 = 1319.46$; $Chi^2 = 30.59$, df = 4 ($P < 0.00001$); $I^2 = 87\%$								
Test for overall effect: Z = 2.20 (P = 0.03)								
**Total (95% CI)**			**191**			**183**	**100.0%**	**39.88 [14.44, 65.32]**

Heterogeneity: $Tau^2 = 948.06$; $Chi^2 = 38.54$, df = 7 ($P < 0.00001$); $I^2 = 82\%$
Test for overall effect: Z = 3.07 (P = 0.002)
Test for subgroup differences: $Chi^2 = 0.02$, df = 1 (P = 0.88), $I^2 = 0\%$

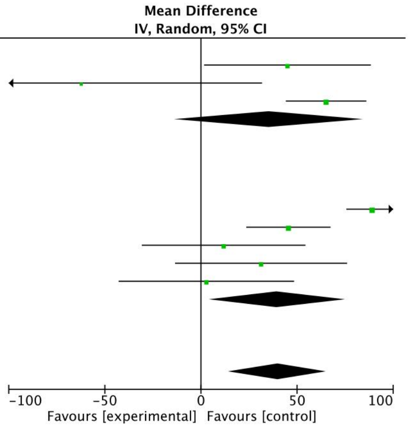

Fig. 3 subgroups analysis to TST (mean age)

Study or Subgroup	Experimental Mean	Experimental SD	Experimental Total	Control Mean	Control SD	Control Total	Weight	Mean Difference IV, Random, 95% CI
**2.3.1 <60y**								
H. Kaynak 2004	428	39	12	383	66	12	11.7%	45.00 [1.63, 88.37]
Hongju Zhang 2013	410.1	24.81	20	321.06	16.97	18	16.9%	89.04 [75.64, 102.44]
P.M.J. Haffmans 1999	297	66.2	3	359.3	58.1	4	5.2%	−62.30 [−156.39, 31.79]
Stein MD 2012	355.9	118.3	63	344.1	118.3	56	11.9%	11.80 [−30.78, 54.38]
Xuemin Li2021	398.87	32.97	31	333.78	50.42	32	15.9%	65.09 [44.12, 86.06]
Z. Lucie 2008	361.9	47	10	359.1	56.7	10	11.3%	2.80 [−42.85, 48.45]
**Subtotal (95% CI)**			**139**			**132**	**72.8%**	**37.97 [4.69, 71.25]**
Heterogeneity: $Tau^2 = 1263.97$; $Chi^2 = 32.33$, df = 5 ($P < 0.00001$); $I^2 = 85\%$								
Test for overall effect: Z = 2.24 (P = 0.03)								
**2.3.2 >60y**								
Lanfang Cao 2018	382.41	45.92	36	336.92	49.61	37	15.7%	45.49 [23.57, 67.41]
W. Jihui 2020	392.48	64.55	16	361.2	60.4	14	11.5%	31.28 [−13.46, 76.02]
**Subtotal (95% CI)**			**52**			**51**	**27.2%**	**42.74 [23.05, 62.42]**
Heterogeneity: $Tau^2 = 0.00$; $Chi^2 = 0.31$, df = 1 (P = 0.58); $I^2 = 0\%$								
Test for overall effect: Z = 4.26 ($P < 0.0001$)								
**Total (95% CI)**			**191**			**183**	**100.0%**	**39.88 [14.44, 65.32]**

Heterogeneity: $Tau^2 = 948.06$; $Chi^2 = 38.54$, df = 7 ($P < 0.00001$); $I^2 = 82\%$
Test for overall effect: Z = 3.07 (P = 0.002)
Test for subgroup differences: $Chi^2 = 0.06$, df = 1 (P = 0.81), $I^2 = 0\%$

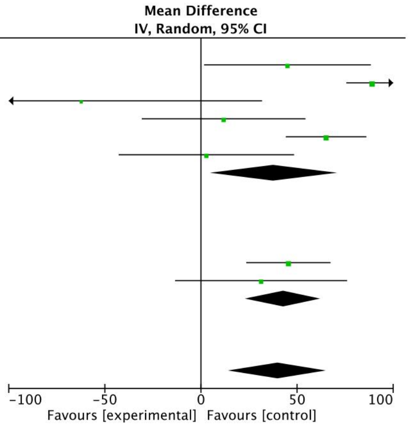

Fig. 4 subgroups analysis to TST(dosage)

Study or Subgroup	Experimental Mean	Experimental SD	Experimental Total	Control Mean	Control SD	Control Total	Weight	Mean Difference IV, Random, 95% CI
**2.4.1 ≤ 50mg/d**								
P.M.J. Haffmans 1999	297	66.2	3	359.3	58.1	4	5.2%	−62.30 [−156.39, 31.79]
Stein MD 2012	355.9	118.3	63	344.1	118.3	56	11.9%	11.80 [−30.78, 54.38]
W. Jihui 2020	392.48	64.55	16	361.2	60.4	14	11.5%	31.28 [−13.46, 76.02]
Xuemin Li 2021	398.87	32.97	31	333.78	50.42	32	15.9%	65.09 [44.12, 86.06]
**Subtotal (95% CI)**			**113**			**106**	**44.4%**	**25.62 [−15.90, 67.14]**
Heterogeneity: $Tau^2 = 1197.46$; $Chi^2 = 11.14$, df = 3 (P = 0.01); $I^2 = 73\%$								
Test for overall effect: Z = 1.21 (P = 0.23)								
**2.4.2 ≥100 mg/d**								
H. Kaynak 2004	428	39	12	383	66	12	11.7%	45.00 [1.63, 88.37]
Hongju Zhang 2013	410.1	24.81	20	321.06	16.97	18	16.9%	89.04 [75.64, 102.44]
Lanfang Cao 2018	382.41	45.92	36	336.92	49.61	37	15.7%	45.49 [23.57, 67.41]
Z. Lucie 2008	361.9	47	10	359.1	56.7	10	11.3%	2.80 [−42.85, 48.45]
**Subtotal (95% CI)**			**78**			**77**	**55.6%**	**49.48 [13.24, 85.72]**
Heterogeneity: $Tau^2 = 1100.67$; $Chi^2 = 22.12$, df = 3 (P < 0.0001); $I^2 = 86\%$								
Test for overall effect: Z = 2.68 (P = 0.007)								
**Total (95% CI)**			**191**			**183**	**100.0%**	**39.88 [14.44, 65.32]**
Heterogeneity: $Tau^2 = 948.06$; $Chi^2 = 38.54$, df = 7 (P < 0.00001); $I^2 = 82\%$								
Test for overall effect: Z = 3.07 (P = 0.002)								
Test for subgroup differences: $Chi^2 = 0.72$, df = 1 (P = 0.40), $I^2 = 0\%$								

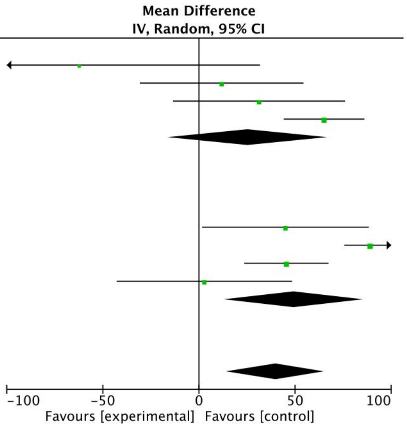

Fig. 5 subgroups analysis to TST(treatment combination or placebo)

Study or Subgroup	Experimental Mean	Experimental SD	Experimental Total	Control Mean	Control SD	Control Total	Weight	Mean Difference IV, Random, 95% CI
**2.3.1 combination with other therapies**								
Lanfang Cao 2018	382.41	45.92	36	336.92	49.61	37	15.7%	45.49 [23.57, 67.41]
Xuemin Li 2021	398.87	32.97	31	333.78	50.42	32	15.9%	65.09 [44.12, 86.06]
Z. Lucie 2008	361.9	47	10	359.1	56.7	10	11.3%	2.80 [−42.85, 48.45]
**Subtotal (95% CI)**			**77**			**79**	**42.8%**	**44.08 [16.29, 71.87]**
Heterogeneity: $Tau^2 = 392.49$; $Chi^2 = 6.25$, df = 2 (P = 0.04); $I^2 = 68\%$								
Test for overall effect: Z = 3.11 (P = 0.002)								
**2.3.2 placebo**								
H. Kaynak 2004	428	39	12	383	66	12	11.7%	45.00 [1.63, 88.37]
Hongju Zhang 2013	410.1	24.81	20	321.06	16.97	18	16.9%	89.04 [75.64, 102.44]
P.M.J. Haffmans 1999	297	66.2	3	359.3	58.1	4	5.2%	−62.30 [−156.39, 31.79]
Stein MD 2012	355.9	118.3	63	344.1	118.3	56	11.9%	11.80 [−30.78, 54.38]
W. Jihui 2020	392.48	64.55	16	361.2	60.4	14	11.5%	31.28 [−13.46, 76.02]
**Subtotal (95% CI)**			**114**			**104**	**57.2%**	**33.36 [−11.48, 78.21]**
Heterogeneity: $Tau^2 = 2017.62$; $Chi^2 = 26.31$, df = 4 (P < 0.0001); $I^2 = 85\%$								
Test for overall effect: Z = 1.46 (P = 0.14)								
**Total (95% CI)**			**191**			**183**	**100.0%**	**39.88 [14.44, 65.32]**
Heterogeneity: $Tau^2 = 948.06$; $Chi^2 = 38.54$, df = 7 (P < 0.00001); $I^2 = 82\%$								
Test for overall effect: Z = 3.07 (P = 0.002)								
Test for subgroup differences: $Chi^2 = 0.16$, df = 1 (P = 0.69), $I^2 = 0\%$								

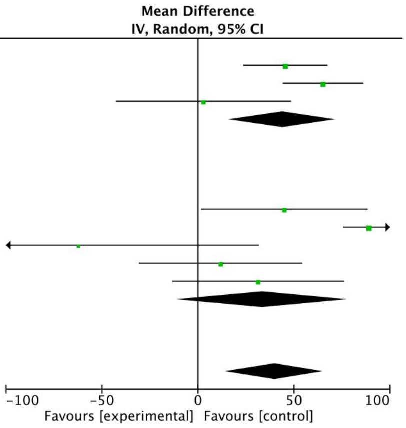

Fig. 6 Funnel plot for TST

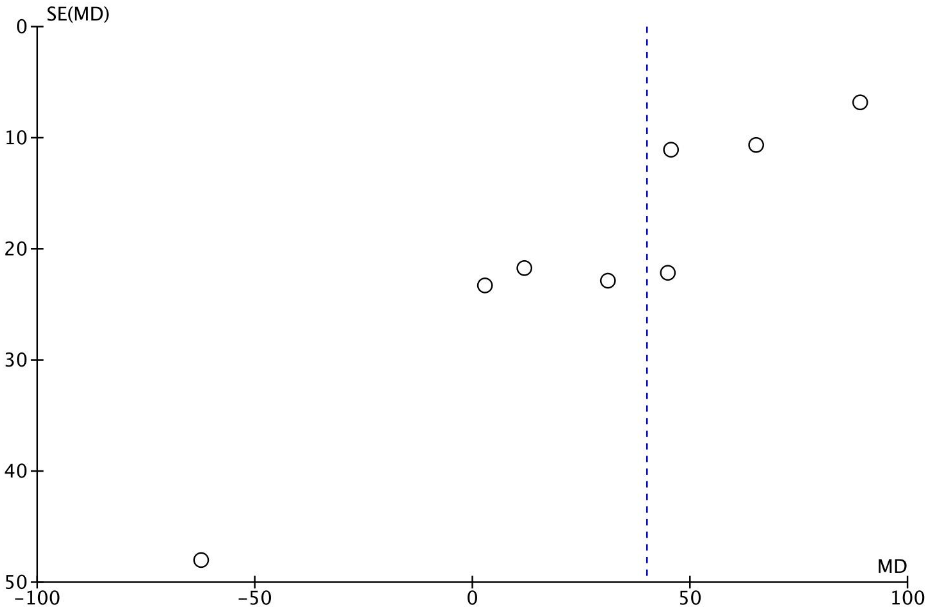

Fig. 7 pooled effects of trazodone on LPS

Study or Subgroup	Experimental Mean	Experimental SD	Experimental Total	Control Mean	Control SD	Control Total	Weight	Mean Difference IV, Random, 95% CI
H. Kaynak 2004	14	14	12	33	53	12	12.1%	−19.00 [−50.02, 12.02]
Hongju Zhang 2013	34.05	2.09	20	73.44	1.92	18	19.0%	−39.39 [−40.67, −38.11]
P.M.J. Haffmans 1999	29.3	19.9	3	50	33.5	4	9.8%	−20.70 [−60.51, 19.11]
Roth AJ 2011	26.2	28.6	16	24.5	18.7	16	16.3%	1.70 [−15.04, 18.44]
W. Jihui 2020	16.82	18.56	16	32.82	16.43	14	17.4%	−16.00 [−28.52, −3.48]
Xuemin Li 2021	21.94	6.96	31	30.72	9.86	32	18.8%	−8.78 [−12.98, −4.58]
Z. Lucie 2008	14.1	17.4	10	65.4	91.1	10	6.5%	−51.30 [−108.78, 6.18]
**Total (95% CI)**			**108**			**106**	**100.0%**	**−19.30 [−37.28, −1.32]**

Heterogeneity: Tau^2 = 442.45; Chi^2 = 219.09, df = 6 (P < 0.00001); I^2 = 97%
Test for overall effect: Z = 2.10 (P = 0.04)

Mean Difference IV, Random, 95% CI

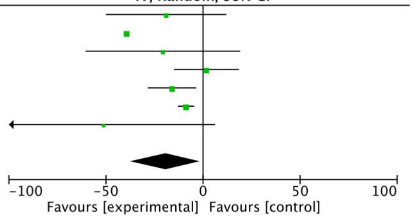

Fig. 8 Sensitivity analysis to LPS

Study or Subgroup	Experimental Mean	Experimental SD	Experimental Total	Control Mean	Control SD	Control Total	Weight	Mean Difference IV, Random, 95% CI
H. Kaynak 2004	14	14	12	33	53	12	3.0%	−19.00 [−50.02, 12.02]
Hongju Zhang 2013	34.05	2.09	20	73.44	1.92	18	0.0%	−39.39 [−40.67, −38.11]
P.M.J. Haffmans 1999	29.3	19.9	3	50	33.5	4	1.9%	−20.70 [−60.51, 19.11]
Roth AJ 2011	26.2	28.6	16	24.5	18.7	16	9.8%	1.70 [−15.04, 18.44]
W. Jihui 2020	16.82	18.56	16	32.82	16.43	14	16.4%	−16.00 [−28.52, −3.48]
Xuemin Li 2021	21.94	6.96	31	30.72	9.86	32	68.0%	−8.78 [−12.98, −4.58]
Z. Lucie 2008	14.1	17.4	10	65.4	91.1	10	0.9%	−51.30 [−108.78, 6.18]
**Total (95% CI)**			**88**			**88**	**100.0%**	**−9.85 [−15.34, −4.37]**

Heterogeneity: Tau^2 = 6.92; Chi^2 = 5.56, df = 5 (P = 0.35); I^2 = 10%
Test for overall effect: Z = 3.52 (P = 0.0004)

Mean Difference IV, Random, 95% CI

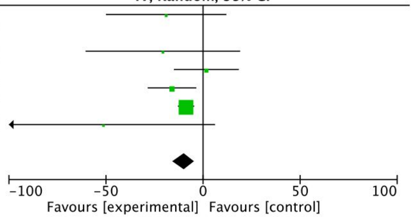

Fig. 9 subgroups analysis to LPS(dosage)

Study or Subgroup	Experimental Mean	Experimental SD	Experimental Total	Control Mean	Control SD	Control Total	Weight	Mean Difference IV, Fixed, 95% CI
**7.3.1 ≥100 mg/d**								
H. Kaynak 2004	14	14	12	33	53	12	0.2%	−19.00 [−50.02, 12.02]
Hongju Zhang 2013	34.05	2.09	20	73.44	1.92	18	90.0%	−39.39 [−40.67, −38.11]
Z. Lucie 2008	14.1	17.4	10	65.4	91.1	10	0.0%	−51.30 [−108.78, 6.18]
**Subtotal (95% CI)**			**42**			**40**	**90.2%**	**−39.36 [−40.64, −38.09]**
Heterogeneity: Chi^2 = 1.82, df = 2 (P = 0.40); I^2 = 0%								
Test for overall effect: Z = 60.57 (P < 0.00001)								
**7.3.2 ≤ 50mg/g**								
P.M.J. Haffmans 1999	29.3	19.9	3	50	33.5	4	0.1%	−20.70 [−60.51, 19.11]
Roth AJ 2011	26.2	28.6	16	24.5	18.7	16	0.5%	1.70 [−15.04, 18.44]
W. Jihui 2020	16.82	18.56	16	32.82	16.43	14	0.9%	−16.00 [−28.52, −3.48]
Xuemin Li 2021	21.94	6.96	31	30.72	9.86	32	8.3%	−8.78 [−12.98, −4.58]
**Subtotal (95% CI)**			**66**			**66**	**9.8%**	**−9.02 [−12.88, −5.16]**
Heterogeneity: Chi^2 = 3.11, df = 3 (P = 0.37); I^2 = 4%								
Test for overall effect: Z = 4.58 (P < 0.00001)								
**Total (95% CI)**			**108**			**106**	**100.0%**	**−36.38 [−37.59, −35.17]**

Heterogeneity: Chi^2 = 219.09, df = 6 (P < 0.00001); I^2 = 97%
Test for overall effect: Z = 58.96 (P < 0.00001)
Test for subgroup differences: Chi^2 = 214.15, df = 1 (P < 0.00001), I^2 = 99.5%

Mean Difference IV, Fixed, 95% CI

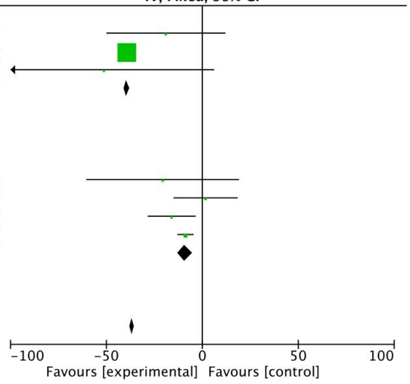

Fig. 10 subgroups analysis to LPS (treatment duration)

Study or Subgroup	Experimental Mean	Experimental SD	Experimental Total	Control Mean	Control SD	Control Total	Weight	Mean Difference IV, Random, 95% CI
**7.2.1 1–2w**								
H. Kaynak 2004	14	14	12	33	53	12	12.1%	−19.00 [−50.02, 12.02]
P.M.J. Haffmans 1999	29.3	19.9	3	50	33.5	4	9.8%	−20.70 [−60.51, 19.11]
Roth AJ 2011	26.2	28.6	16	24.5	18.7	16	16.3%	1.70 [−15.04, 18.44]
Xuemin Li 2021	21.94	6.96	31	30.72	9.86	32	18.8%	−8.78 [−12.98, −4.58]
**Subtotal (95% CI)**			**62**			**64**	**57.1%**	**−8.47 [−12.49, −4.45]**
Heterogeneity: Tau^2 = 0.00; Chi^2 = 2.24, df = 3 (P = 0.52); I^2 = 0%								
Test for overall effect: Z = 4.13 (P < 0.0001)								
**7.2.2 ≥1m**								
Hongju Zhang 2013	34.05	2.09	20	73.44	1.92	18	19.0%	−39.39 [−40.67, −38.11]
W. Jihui 2020	16.82	18.56	16	32.82	16.43	14	17.4%	−16.00 [−28.52, −3.48]
Z. Lucie 2008	14.1	17.4	10	65.4	91.1	10	6.5%	−51.30 [−108.78, 6.18]
**Subtotal (95% CI)**			**46**			**42**	**42.9%**	**−30.95 [−51.48, −10.41]**
Heterogeneity: Tau^2 = 225.17; Chi^2 = 13.44, df = 2 (P = 0.001); I^2 = 85%								
Test for overall effect: Z = 2.95 (P = 0.003)								
**Total (95% CI)**			**108**			**106**	**100.0%**	**−19.30 [−37.28, −1.32]**

Heterogeneity: Tau^2 = 442.45; Chi^2 = 219.09, df = 6 (P < 0.00001); I^2 = 97%
Test for overall effect: Z = 2.10 (P = 0.04)
Test for subgroup differences: Chi^2 = 4.43, df = 1 (P = 0.04), I^2 = 77.4%

Mean Difference IV, Random, 95% CI

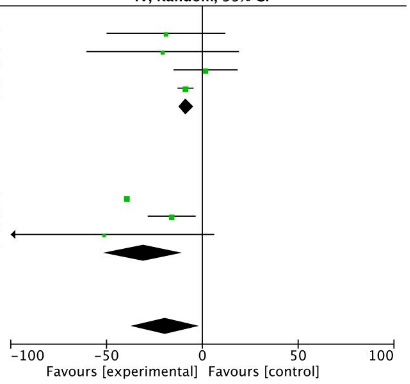

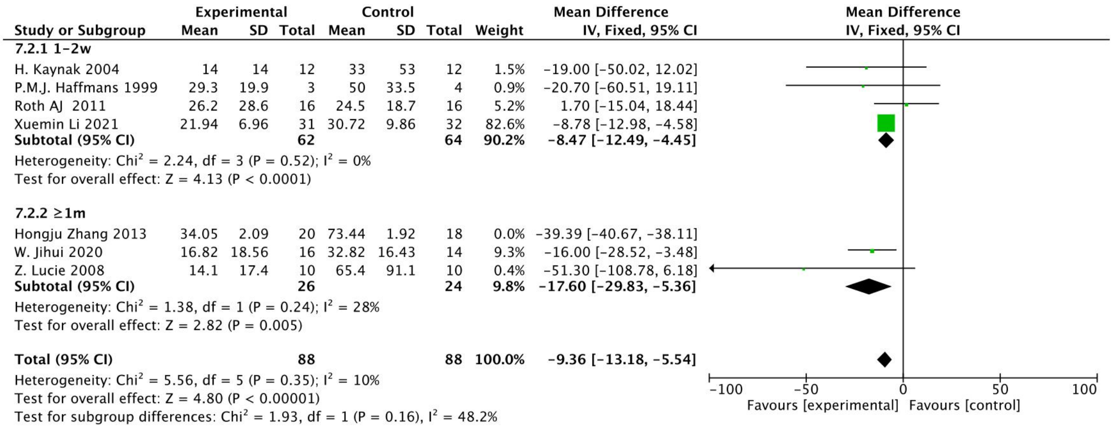

Study or Subgroup	Experimental Mean	Experimental SD	Experimental Total	Control Mean	Control SD	Control Total	Weight	Mean Difference IV, Fixed, 95% CI
**7.2.1 1–2w**								
H. Kaynak 2004	14	14	12	33	53	12	1.5%	−19.00 [−50.02, 12.02]
P.M.J. Haffmans 1999	29.3	19.9	3	50	33.5	4	0.9%	−20.70 [−60.51, 19.11]
Roth AJ 2011	26.2	28.6	16	24.5	18.7	16	5.2%	1.70 [−15.04, 18.44]
Xuemin Li 2021	21.94	6.96	31	30.72	9.86	32	82.6%	−8.78 [−12.98, −4.58]
**Subtotal (95% CI)**			**62**			**64**	**90.2%**	**−8.47 [−12.49, −4.45]**
Heterogeneity: Chi^2 = 2.24, df = 3 (P = 0.52); I^2 = 0%								
Test for overall effect: Z = 4.13 (P < 0.0001)								
**7.2.2 ≥1m**								
Hongju Zhang 2013	34.05	2.09	20	73.44	1.92	18	0.0%	−39.39 [−40.67, −38.11]
W. Jihui 2020	16.82	18.56	16	32.82	16.43	14	9.3%	−16.00 [−28.52, −3.48]
Z. Lucie 2008	14.1	17.4	10	65.4	91.1	10	0.4%	−51.30 [−108.78, 6.18]
**Subtotal (95% CI)**			**26**			**24**	**9.8%**	**−17.60 [−29.83, −5.36]**
Heterogeneity: Chi^2 = 1.38, df = 1 (P = 0.24); I^2 = 28%								
Test for overall effect: Z = 2.82 (P = 0.005)								
**Total (95% CI)**			**88**			**88**	**100.0%**	**−9.36 [−13.18, −5.54]**
Heterogeneity: Chi^2 = 5.56, df = 5 (P = 0.35); I^2 = 10%								
Test for overall effect: Z = 4.80 (P < 0.00001)								
Test for subgroup differences: Chi^2 = 1.93, df = 1 (P = 0.16), I^2 = 48.2%								

Fig. 11 subgroups analysis to LPS(combination with other therapies or placebo)

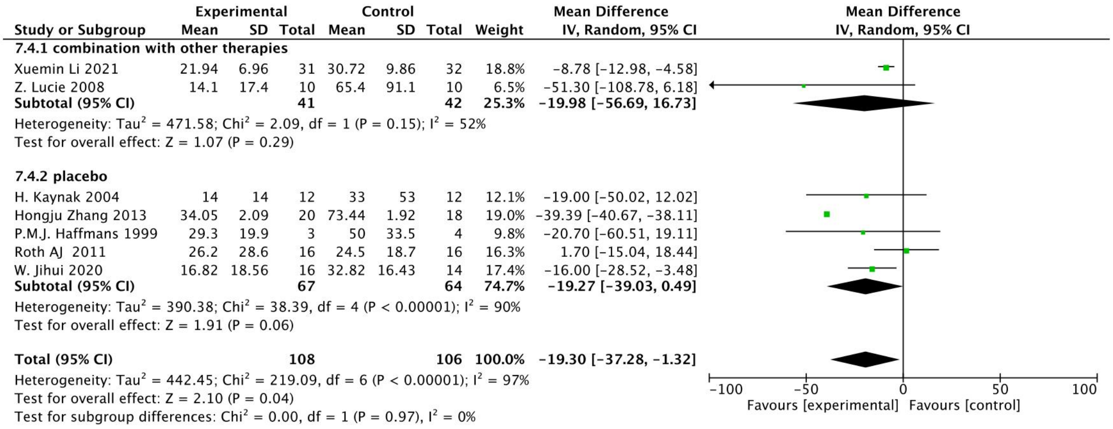

Study or Subgroup	Experimental Mean	Experimental SD	Experimental Total	Control Mean	Control SD	Control Total	Weight	Mean Difference IV, Random, 95% CI
**7.4.1 combination with other therapies**								
Xuemin Li 2021	21.94	6.96	31	30.72	9.86	32	18.8%	−8.78 [−12.98, −4.58]
Z. Lucie 2008	14.1	17.4	10	65.4	91.1	10	6.5%	−51.30 [−108.78, 6.18]
**Subtotal (95% CI)**			**41**			**42**	**25.3%**	**−19.98 [−56.69, 16.73]**
Heterogeneity: Tau^2 = 471.58; Chi^2 = 2.09, df = 1 (P = 0.15); I^2 = 52%								
Test for overall effect: Z = 1.07 (P = 0.29)								
**7.4.2 placebo**								
H. Kaynak 2004	14	14	12	33	53	12	12.1%	−19.00 [−50.02, 12.02]
Hongju Zhang 2013	34.05	2.09	20	73.44	1.92	18	19.0%	−39.39 [−40.67, −38.11]
P.M.J. Haffmans 1999	29.3	19.9	3	50	33.5	4	9.8%	−20.70 [−60.51, 19.11]
Roth AJ 2011	26.2	28.6	16	24.5	18.7	16	16.3%	1.70 [−15.04, 18.44]
W. Jihui 2020	16.82	18.56	16	32.82	16.43	14	17.4%	−16.00 [−28.52, −3.48]
**Subtotal (95% CI)**			**67**			**64**	**74.7%**	**−19.27 [−39.03, 0.49]**
Heterogeneity: Tau^2 = 390.38; Chi^2 = 38.39, df = 4 (P < 0.00001); I^2 = 90%								
Test for overall effect: Z = 1.91 (P = 0.06)								
**Total (95% CI)**			**108**			**106**	**100.0%**	**−19.30 [−37.28, −1.32]**
Heterogeneity: Tau^2 = 442.45; Chi^2 = 219.09, df = 6 (P < 0.00001); I^2 = 97%								
Test for overall effect: Z = 2.10 (P = 0.04)								
Test for subgroup differences: Chi^2 = 0.00, df = 1 (P = 0.97), I^2 = 0%								

Fig. 12 Funnel plot for LPS

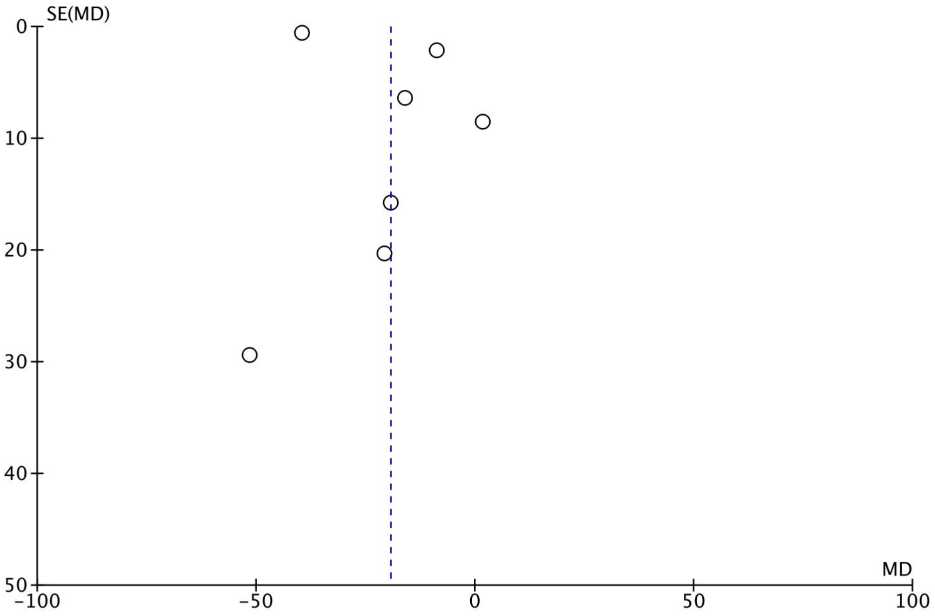

Fig. 13 pooled effects of trazodone on SE

Study or Subgroup	Experimental Mean	Experimental SD	Experimental Total	Control Mean	Control SD	Control Total	Weight	Mean Difference IV, Random, 95% CI
Hongju Zhang 2013	75	2	20	46	3	18	20.9%	29.00 [27.36, 30.64]
Roth AJ 2011	85.3	11	16	81.7	12	16	20.0%	3.60 [−4.38, 11.58]
Stein MD 2012	85.8	12.1	63	85.3	12.1	56	20.7%	0.50 [−3.86, 4.86]
W. Jihui 2020	81.67	11.87	16	75.88	22.4	14	18.6%	5.79 [−7.31, 18.89]
Z. Lucie 2008	80.8	11.5	10	80.9	8	10	19.8%	−0.10 [−8.78, 8.58]
**Total (95% CI)**			**125**			**114**	**100.0%**	**7.94 [−8.92, 24.81]**

Heterogeneity: $Tau^2 = 353.60$; $Chi^2 = 209.56$, df = 4 (P < 0.00001); $I^2 = 98\%$
Test for overall effect: Z = 0.92 (P = 0.36)

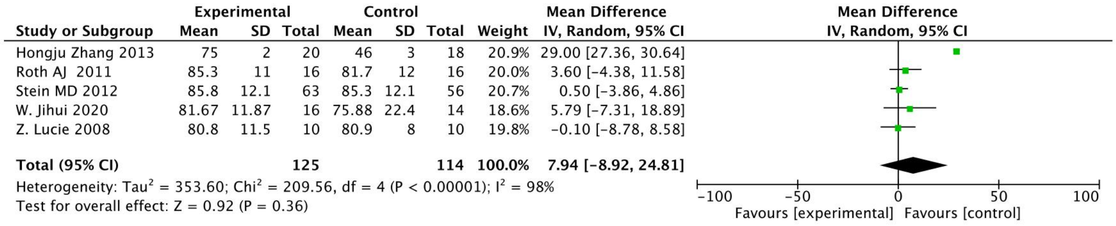

Fig. 14 Sensitivity analysis to SE

Study or Subgroup	Experimental Mean	Experimental SD	Experimental Total	Control Mean	Control SD	Control Total	Weight	Mean Difference IV, Fixed, 95% CI
Hongju Zhang 2013	75	2	20	46	3	18	0.0%	29.00 [27.36, 30.64]
Roth AJ 2011	85.3	11	16	81.7	12	16	18.0%	3.60 [−4.38, 11.58]
Stein MD 2012	85.8	12.1	63	85.3	12.1	56	60.2%	0.50 [−3.86, 4.86]
W. Jihui 2020	81.67	11.87	16	75.88	22.4	14	6.7%	5.79 [−7.31, 18.89]
Z. Lucie 2008	80.8	11.5	10	80.9	8	10	15.2%	−0.10 [−8.78, 8.58]
**Total (95% CI)**			**105**			**96**	**100.0%**	**1.32 [−2.06, 4.70]**

Heterogeneity: $Chi^2 = 1.00$, df = 3 (P = 0.80); $I^2 = 0\%$
Test for overall effect: Z = 0.76 (P = 0.44)

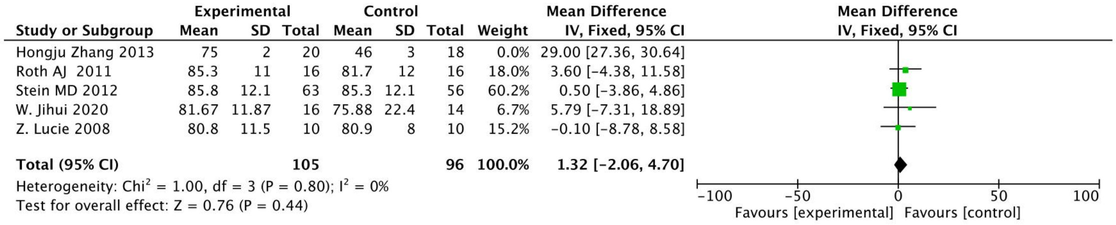

Fig. 15 Funnel plot for SE

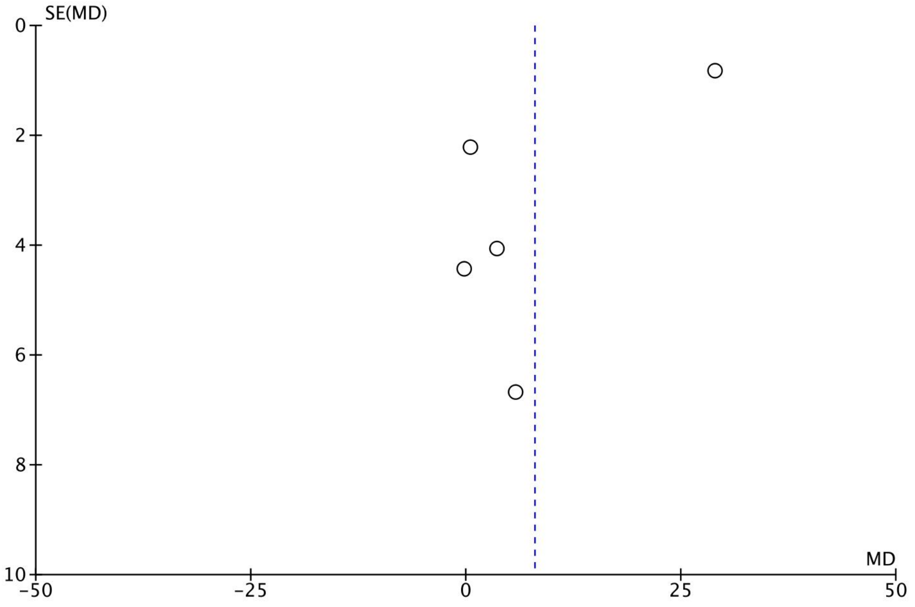

Fig. 16 pooled effects of trazodone on WASO

Study or Subgroup	Experimental Mean	Experimental SD	Experimental Total	Control Mean	Control SD	Control Total	Weight	Std. Mean Difference IV, Fixed, 95% CI
Le Bon O 2003	3	6.49	8	12	6.49	8	12.5%	−1.31 [−2.42, −0.20]
P.M.J. Haffmans 1999	36.5	35.1	3	47.5	31.6	4	6.7%	−0.28 [−1.79, 1.23]
Roth AJ 2011	52.9	54.9	16	74.3	61.1	16	31.5%	−0.36 [−1.06, 0.34]
W. Jihui 2020	42.42	20.88	16	52.46	25.87	14	29.2%	−0.42 [−1.14, 0.31]
Z. Lucie 2008	74.8	56.7	10	75	33.2	10	20.1%	−0.00 [−0.88, 0.87]
**Total (95% CI)**			**53**			**52**	**100.0%**	**−0.42 [−0.81, −0.03]**

Heterogeneity: $Chi^2 = 3.40$, df = 4 (P = 0.49); $I^2 = 0\%$
Test for overall effect: Z = 2.09 (P = 0.04)

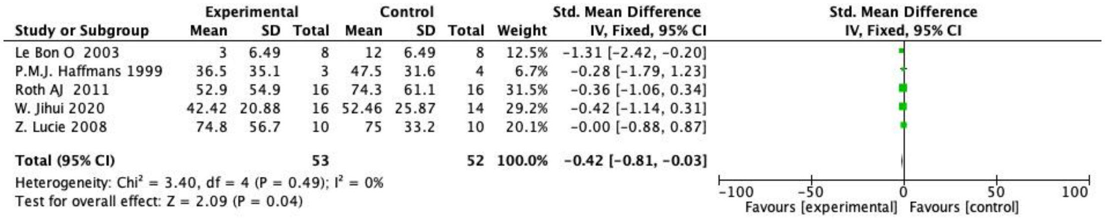

Fig. 17 Sensitivity analysis of WASO to combination of treatment

Study or Subgroup	Experimental Mean	SD	Total	Control Mean	SD	Total	Weight	Std. Mean Difference IV, Fixed, 95% CI
Le Bon O 2003	3	6.49	8	12	6.49	8	15.6%	−1.31 [−2.42, −0.20]
P.M.J. Haffmans 1999	36.5	35.1	3	47.5	31.6	4	8.4%	−0.28 [−1.79, 1.23]
Roth AJ 2011	52.9	54.9	16	74.3	61.1	16	39.4%	−0.36 [−1.06, 0.34]
W. Jihui 2020	42.42	20.88	16	52.46	25.87	14	36.5%	−0.42 [−1.14, 0.31]
Z. Lucie 2008	74.8	56.7	10	75	33.2	10	0.0%	−0.00 [−0.88, 0.87]
**Total (95% CI)**			**43**			**42**	**100.0%**	**−0.52 [−0.96, −0.08]**

Heterogeneity: $Chi^2 = 2.32$, df = 3 (P = 0.51); $I^2 = 0\%$
Test for overall effect: Z = 2.33 (P = 0.02)

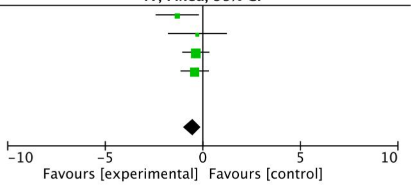

Fig. 18 Funnel plot for WASO

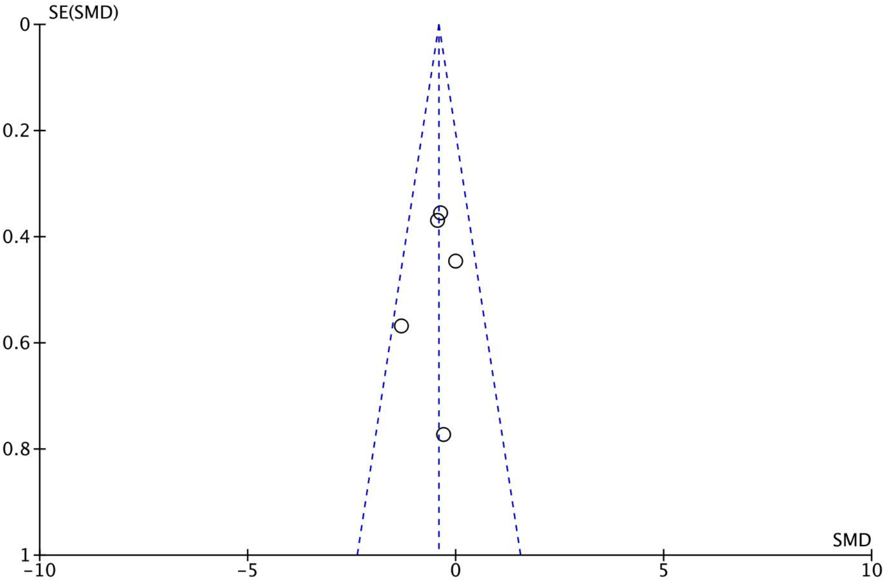

Fig. 19 pooled effects of trazodone on NAs (including both arousal times and arousal index)

Study or Subgroup	Experimental Mean	SD	Total	Control Mean	SD	Total	Weight	Std. Mean Difference IV, Fixed, 95% CI
H. Kaynak 2004	12	13	12	30	18	12	8.0%	−1.11 [−1.98, −0.24]
P.M.J. Haffmans 1999	2.3	2.2	3	5.3	3.4	4	2.3%	−0.85 [−2.49, 0.79]
Roth AJ 2011	12.7	6.5	16	20	9.9	16	11.5%	−0.85 [−1.58, −0.12]
Stein MD 2012	8.8	1.9	63	9.5	1.9	56	46.0%	−0.37 [−0.73, −0.00]
W. Jihui 2020	22.51	11.57	16	26.37	11.1	14	11.6%	−0.33 [−1.05, 0.39]
Xuemin Li2021	2.06	0.61	31	3.16	1.08	32	20.7%	−1.23 [−1.78, −0.69]
**Total (95% CI)**			**141**			**134**	**100.0%**	**−0.67 [−0.91, −0.42]**

Heterogeneity: $Chi^2 = 8.94$, df = 5 (P = 0.11); $I^2 = 44\%$
Test for overall effect: Z = 5.31 (P < 0.00001)

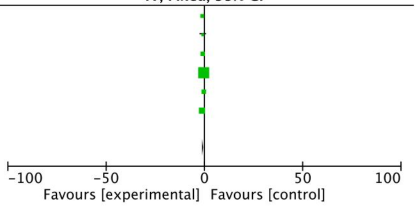

Fig. 20 Sensitivity analysis of NAs to combination of treatment

Study or Subgroup	Experimental Mean	SD	Total	Control Mean	SD	Total	Weight	Std. Mean Difference IV, Fixed, 95% CI
H. Kaynak 2004	12	13	12	30	18	12	10.1%	−1.11 [−1.98, −0.24]
P.M.J. Haffmans 1999	2.3	2.2	3	5.3	3.4	4	2.8%	−0.85 [−2.49, 0.79]
Roth AJ 2011	12.7	6.5	16	20	9.9	16	14.4%	−0.85 [−1.58, −0.12]
Stein MD 2012	8.8	1.9	63	9.5	1.9	56	58.0%	−0.37 [−0.73, −0.00]
W. Jihui 2020	22.51	11.57	16	26.37	11.1	14	14.6%	−0.33 [−1.05, 0.39]
Xuemin Li2021	2.06	0.61	31	3.16	1.08	32	0.0%	−1.23 [−1.78, −0.69]
**Total (95% CI)**			**110**			**102**	**100.0%**	**−0.52 [−0.80, −0.24]**

Heterogeneity: $Chi^2 = 3.64$, df = 4 (P = 0.46); $I^2 = 0\%$
Test for overall effect: Z = 3.68 (P = 0.0002)

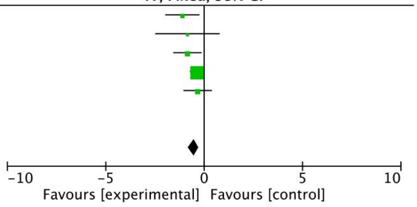

Fig. 21 Funnel plot for NAs

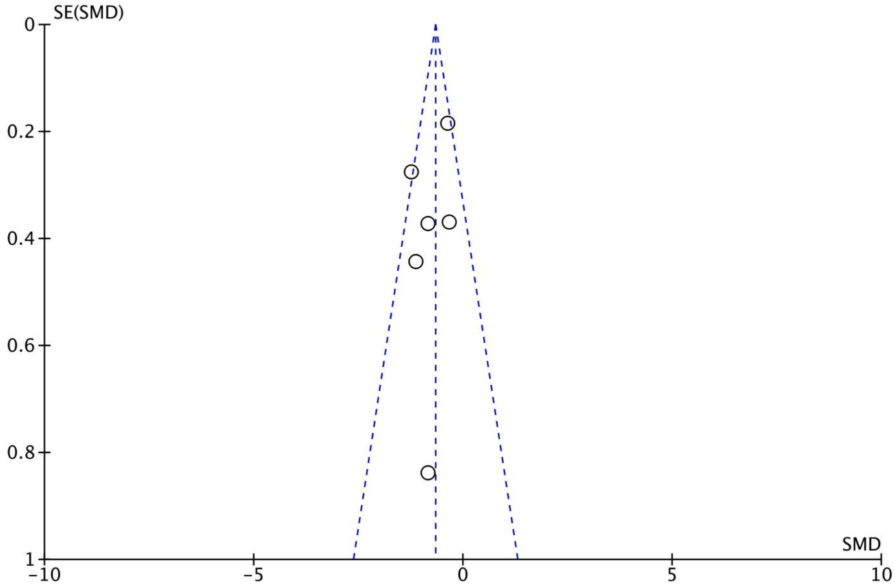

Fig. 22 pooled effects of trazodone on AHI

Study or Subgroup	Experimental Mean	Experimental SD	Experimental Total	Control Mean	Control SD	Control Total	Weight	Mean Difference IV, Random, 95% CI
C.-Y. Chen 2021	25.4	15.4	16	39.1	18.4	14	30.6%	−13.70 [−25.94, −1.46]
Stein MD 2012	2.8	44.6	22	2	44.6	22	11.2%	0.80 [−25.56, 27.16]
W. Jihui 2020	3.92	1.72	63	4.12	1.35	56	58.2%	−0.20 [−0.75, 0.35]
**Total (95% CI)**			**101**			**92**	**100.0%**	**−4.21 [−14.02, 5.59]**

Heterogeneity: $Tau^2 = 42.90$; $Chi^2 = 4.67$, df = 2 (P = 0.10); $I^2 = 57\%$
Test for overall effect: Z = 0.84 (P = 0.40)

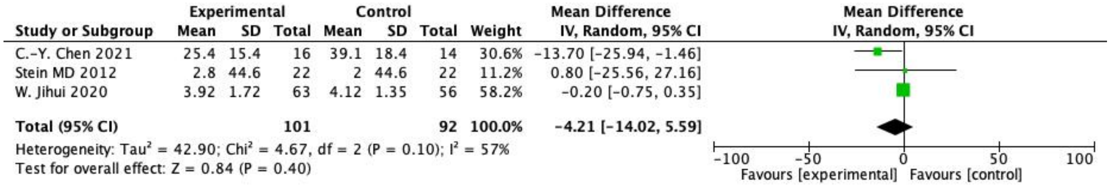

Fig. 23 pooled effects of trazodone on REML

Study or Subgroup	Experimental Mean	Experimental SD	Experimental Total	Control Mean	Control SD	Control Total	Weight	Mean Difference IV, Fixed, 95% CI
H. Kaynak 2004	230	91	12	244	122	12	12.0%	−14.00 [−100.11, 72.11]
P.M.J. Haffmans 1999	193.5	115.9	3	257.7	27.1	4	5.0%	−64.20 [−198.01, 69.61]
Roth AJ 2011	93	53.1	16	84.3	40.8	16	83.0%	8.70 [−24.11, 41.51]
**Total (95% CI)**			**31**			**32**	**100.0%**	**2.33 [−27.56, 32.22]**

Heterogeneity: $Chi^2 = 1.23$, df = 2 (P = 0.54); $I^2 = 0\%$
Test for overall effect: Z = 0.15 (P = 0.88)

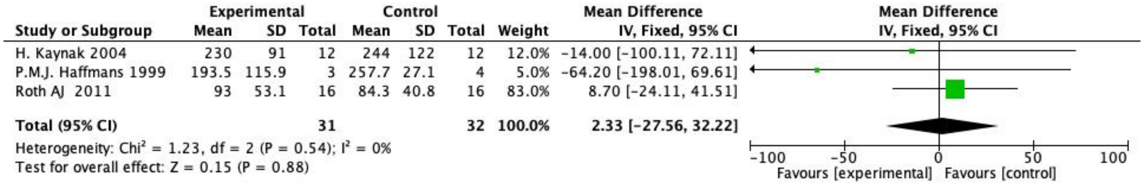

Fig. 24 pooled effects of trazodone on REM

Study or Subgroup	Experimental Mean	Experimental SD	Experimental Total	Control Mean	Control SD	Control Total	Weight	Std. Mean Difference IV, Random, 95% CI
H. Kaynak 2004	16	8	12	15	6	12	13.4%	0.14 [−0.66, 0.94]
Lanfang Cao 2018	106.73	21.09	36	82.19	14.76	37	17.3%	1.34 [0.83, 1.85]
P.M.J. Haffmans 1999	14	15.6	3	11.7	2.5	4	6.9%	0.19 [−1.31, 1.70]
Roth AJ 2011	19.1	4.7	16	19.9	5.4	37	16.3%	−0.15 [−0.74, 0.44]
Stein MD 2012	19.9	11.11	63	17.9	11.11	56	19.2%	0.18 [−0.18, 0.54]
W. Jihui 2020	15	7.22	16	14.93	7.15	14	14.5%	0.01 [−0.71, 0.73]
Z. Lucie 2008	65	26.8	10	83	47.9	10	12.4%	−0.44 [−1.33, 0.45]
**Total (95% CI)**			**156**			**170**	**100.0%**	**0.22 [−0.26, 0.70]**

Heterogeneity: $Tau^2 = 0.28$; $Chi^2 = 22.08$, df = 6 (P = 0.001); $I^2 = 73\%$
Test for overall effect: Z = 0.90 (P = 0.37)

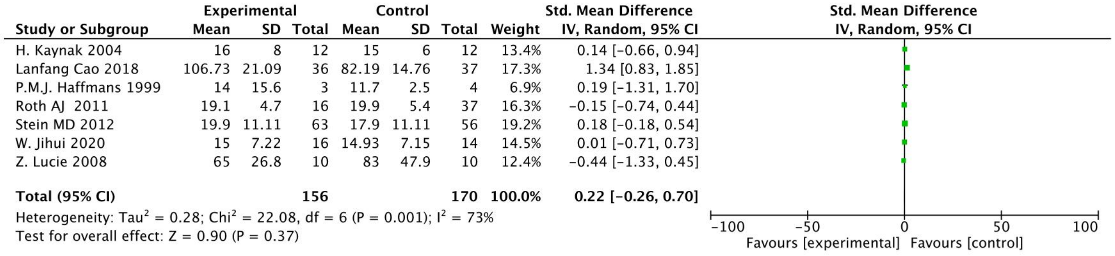

Fig. 25 Sensitivity analysis to REM

Study or Subgroup	Experimental Mean	Experimental SD	Experimental Total	Control Mean	Control SD	Control Total	Weight	Std. Mean Difference IV, Fixed, 95% CI
H. Kaynak 2004	16	8	12	15	6	12	9.9%	0.14 [−0.66, 0.94]
Lanfang Cao 2018	106.73	21.09	36	82.19	14.76	37	0.0%	1.34 [0.83, 1.85]
P.M.J. Haffmans 1999	14	15.6	3	11.7	2.5	4	2.8%	0.19 [−1.31, 1.70]
Roth AJ 2011	19.1	4.7	16	19.9	5.4	37	18.4%	−0.15 [−0.74, 0.44]
Stein MD 2012	19.9	11.11	63	17.9	11.11	56	48.7%	0.18 [−0.18, 0.54]
W. Jihui 2020	15	7.22	16	14.93	7.15	14	12.3%	0.01 [−0.71, 0.73]
Z. Lucie 2008	65	26.8	10	83	47.9	10	8.0%	−0.44 [−1.33, 0.45]
**Total (95% CI)**			**120**			**133**	**100.0%**	**0.04 [−0.21, 0.30]**

Heterogeneity: $Chi^2 = 2.22$, df = 5 (P = 0.82); $I^2 = 0\%$
Test for overall effect: Z = 0.34 (P = 0.73)

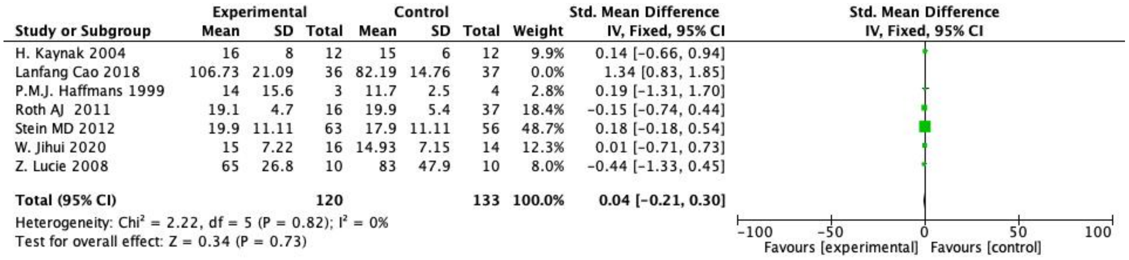

Fig. 26 subgroups analysis to REM (treatment duration)

Study or Subgroup	Experimental Mean	Experimental SD	Experimental Total	Control Mean	Control SD	Control Total	Weight	Std. Mean Difference IV, Random, 95% CI
**3.2.1 1w**								
H. Kaynak 2004	16	8	12	15	6	12	13.4%	0.14 [−0.66, 0.94]
P.M.J. Haffmans 1999	14	15.6	3	11.7	2.5	4	6.9%	0.19 [−1.31, 1.70]
Roth AJ 2011	19.1	4.7	16	19.9	5.4	37	16.3%	−0.15 [−0.74, 0.44]
**Subtotal (95% CI)**			**31**			**53**	**36.6%**	**−0.03 [−0.48, 0.42]**
Heterogeneity: $Tau^2 = 0.00$; $Chi^2 = 0.41$, df = 2 (P = 0.81); $I^2 = 0\%$								
Test for overall effect: Z = 0.13 (P = 0.90)								
**3.2.2 ≥1m**								
Lanfang Cao 2018	106.73	21.09	36	82.19	14.76	37	17.3%	1.34 [0.83, 1.85]
Stein MD 2012	19.9	11.11	63	17.9	11.11	56	19.2%	0.18 [−0.18, 0.54]
W. Jihui 2020	15	7.22	16	14.93	7.15	14	14.5%	0.01 [−0.71, 0.73]
Z. Lucie 2008	65	26.8	10	83	47.9	10	12.4%	−0.44 [−1.33, 0.45]
**Subtotal (95% CI)**			**125**			**117**	**63.4%**	**0.32 [−0.41, 1.04]**
Heterogeneity: $Tau^2 = 0.45$; $Chi^2 = 19.00$, df = 3 (P = 0.0003); $I^2 = 84\%$								
Test for overall effect: Z = 0.86 (P = 0.39)								
**Total (95% CI)**			**156**			**170**	**100.0%**	**0.22 [−0.26, 0.70]**

Heterogeneity: $Tau^2 = 0.28$; $Chi^2 = 22.08$, df = 6 (P = 0.001); $I^2 = 73\%$
Test for overall effect: Z = 0.90 (P = 0.37)
Test for subgroup differences: $Chi^2 = 0.63$, df = 1 (P = 0.43), $I^2 = 0\%$

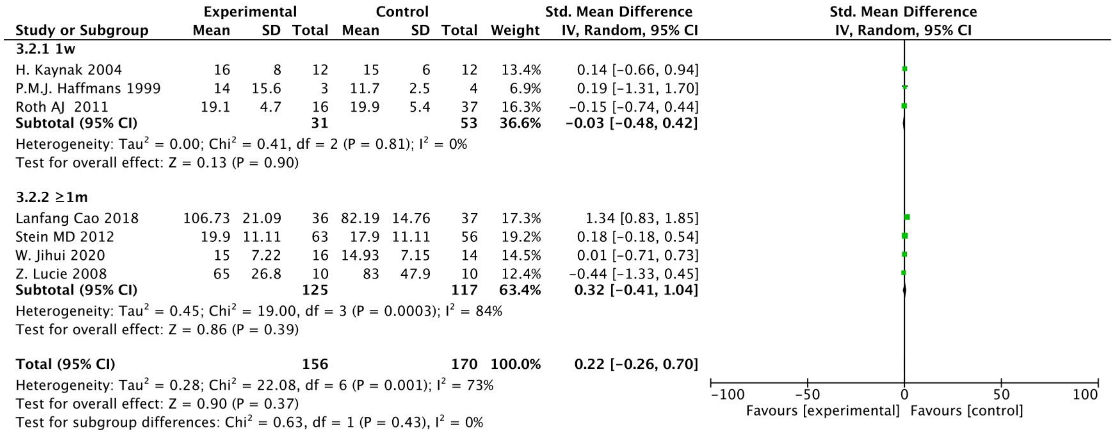

Fig. 27 subgroups analysis to REM(dosage)

Study or Subgroup	Experimental Mean	Experimental SD	Experimental Total	Control Mean	Control SD	Control Total	Weight	Std. Mean Difference IV, Random, 95% CI
**3.3.1 dose≥100 mg/d**								
H. Kaynak 2004	16	8	12	15	6	12	13.4%	0.14 [−0.66, 0.94]
Lanfang Cao 2018	106.73	21.09	36	82.19	14.76	37	17.3%	1.34 [0.83, 1.85]
Z. Lucie 2008	65	26.8	10	83	47.9	10	12.4%	−0.44 [−1.33, 0.45]
**Subtotal (95% CI)**			**58**			**59**	**43.1%**	**0.39 [−0.73, 1.50]**
Heterogeneity: $Tau^2 = 0.83$; $Chi^2 = 14.23$, df = 2 (P = 0.0008); $I^2 = 86\%$								
Test for overall effect: Z = 0.68 (P = 0.49)								
**3.3.2 dose≤ 50mg/d**								
P.M.J. Haffmans 1999	14	15.6	3	11.7	2.5	4	6.9%	0.19 [−1.31, 1.70]
Roth AJ 2011	19.1	4.7	16	19.9	5.4	37	16.3%	−0.15 [−0.74, 0.44]
Stein MD 2012	19.9	11.11	63	17.9	11.11	56	19.2%	0.18 [−0.18, 0.54]
W. Jihui 2020	15	7.22	16	14.93	7.15	14	14.5%	0.01 [−0.71, 0.73]
**Subtotal (95% CI)**			**98**			**111**	**56.9%**	**0.08 [−0.20, 0.36]**
Heterogeneity: $Tau^2 = 0.00$; $Chi^2 = 0.94$, df = 3 (P = 0.81); $I^2 = 0\%$								
Test for overall effect: Z = 0.57 (P = 0.57)								
**Total (95% CI)**			**156**			**170**	**100.0%**	**0.22 [−0.26, 0.70]**

Heterogeneity: $Tau^2 = 0.28$; $Chi^2 = 22.08$, df = 6 (P = 0.001); $I^2 = 73\%$
Test for overall effect: Z = 0.90 (P = 0.37)
Test for subgroup differences: $Chi^2 = 0.28$, df = 1 (P = 0.60), $I^2 = 0\%$

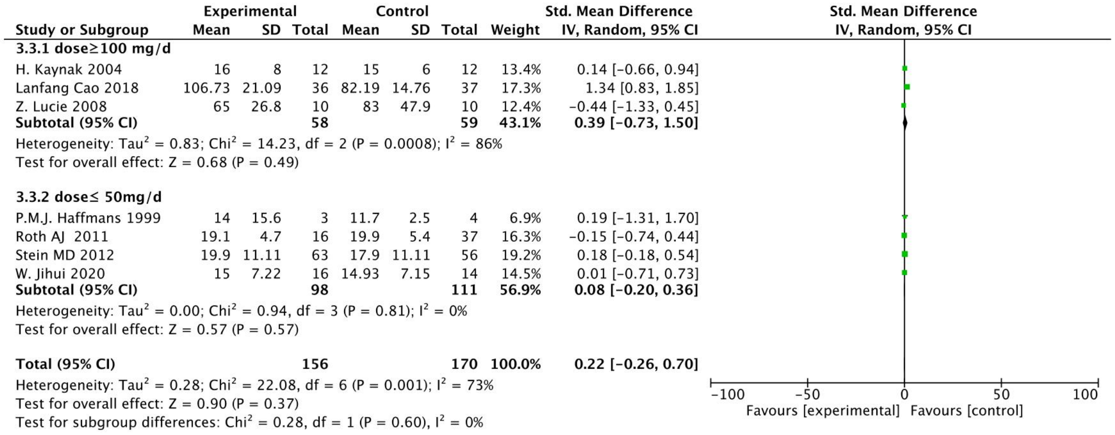

Fig. 28 subgroups analysis to REM (treatment combination or placebo)

Study or Subgroup	Experimental Mean	Experimental SD	Experimental Total	Control Mean	Control SD	Control Total	Weight	Std. Mean Difference IV, Random, 95% CI
**3.4.1 combination with other therapies**								
Lanfang Cao 2018	106.73	21.09	36	82.19	14.76	37	17.3%	1.34 [0.83, 1.85]
Z. Lucie 2008	65	26.8	10	83	47.9	10	12.4%	−0.44 [−1.33, 0.45]
**Subtotal (95% CI)**			**46**			**47**	**29.7%**	**0.49 [−1.26, 2.23]**
Heterogeneity: Tau^2 = 1.45; Chi^2 = 11.59, df = 1 (P = 0.0007); I^2 = 91%								
Test for overall effect: Z = 0.55 (P = 0.59)								
**3.4.2 Placebo**								
H. Kaynak 2004	16	8	12	15	6	12	13.4%	0.14 [−0.66, 0.94]
P.M.J. Haffmans 1999	14	15.6	3	11.7	2.5	4	6.9%	0.19 [−1.31, 1.70]
Roth AJ 2011	19.1	4.7	16	19.9	5.4	37	16.3%	−0.15 [−0.74, 0.44]
Stein MD 2012	19.9	11.11	63	17.9	11.11	56	19.2%	0.18 [−0.18, 0.54]
W. Jihui 2020	15	7.22	16	14.93	7.15	14	14.5%	0.01 [−0.71, 0.73]
**Subtotal (95% CI)**			**110**			**123**	**70.3%**	**0.09 [−0.18, 0.35]**
Heterogeneity: Tau^2 = 0.00; Chi^2 = 0.96, df = 4 (P = 0.92); I^2 = 0%								
Test for overall effect: Z = 0.64 (P = 0.52)								
**Total (95% CI)**			**156**			**170**	**100.0%**	**0.22 [−0.26, 0.70]**
Heterogeneity: Tau^2 = 0.28; Chi^2 = 22.08, df = 6 (P = 0.001); I^2 = 73%								
Test for overall effect: Z = 0.90 (P = 0.37)								
Test for subgroup differences: Chi^2 = 0.20, df = 1 (P = 0.66), I^2 = 0%								

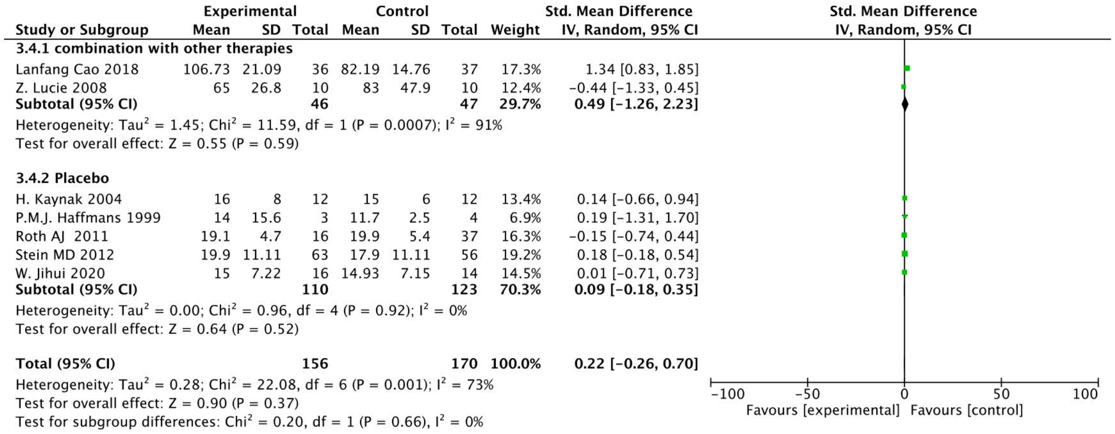

Fig. 29 Funnel plot for REM

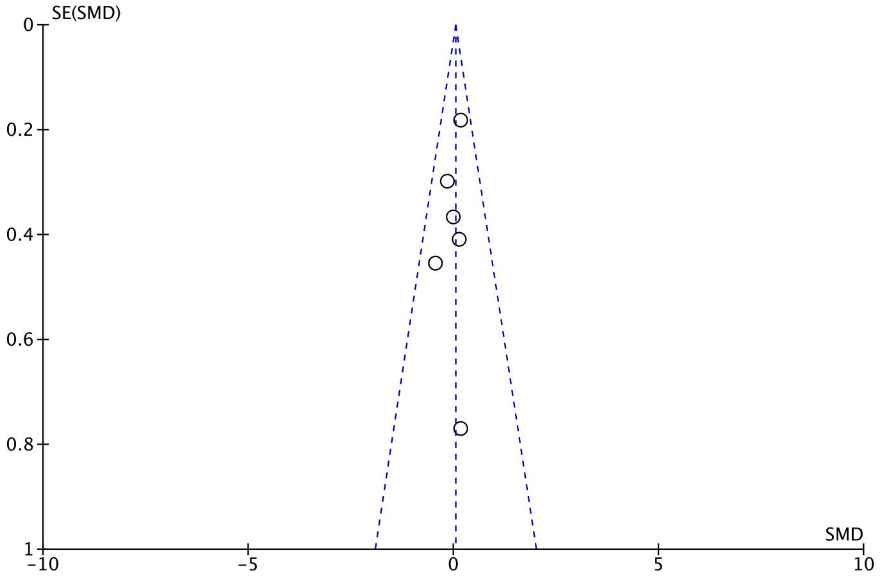

Fig. 30 pooled effects of trazodone on N1

Study or Subgroup	Experimental Mean	Experimental SD	Experimental Total	Control Mean	Control SD	Control Total	Weight	Std. Mean Difference IV, Random, 95% CI
H. Kaynak 2004	3	2	12	7	3	12	14.8%	−1.51 [−2.44, −0.59]
P.M.J. Haffmans 1999	7.9	4.9	3	7.6	4.2	4	8.2%	0.06 [−1.44, 1.55]
Roth AJ 2011	17.9	9.5	16	28.8	11.6	16	18.0%	−1.00 [−1.74, −0.26]
Stein MD 2012	2.4	2.5	63	2.5	2.5	56	25.7%	−0.04 [−0.40, 0.32]
W. Jihui 2020	9.44	4.96	16	13.12	5.24	14	18.0%	−0.70 [−1.45, 0.04]
Z. Lucie 2008	28.5	11.1	10	38.5	21	10	15.3%	−0.57 [−1.47, 0.33]
**Total (95% CI)**			**120**			**112**	**100.0%**	**−0.62 [−1.13, −0.12]**
Heterogeneity: Tau^2 = 0.23; Chi^2 = 13.06, df = 5 (P = 0.02); I^2 = 62%								
Test for overall effect: Z = 2.42 (P = 0.02)								

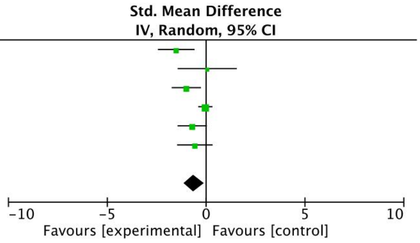

Fig. 31 Sensitivity analysis of heterogeneity to N1

Study or Subgroup	Experimental Mean	Experimental SD	Experimental Total	Control Mean	Control SD	Control Total	Weight	Std. Mean Difference IV, Fixed, 95% CI
H. Kaynak 2004	3	2	12	7	3	12	17.9%	−1.51 [−2.44, −0.59]
P.M.J. Haffmans 1999	7.9	4.9	3	7.6	4.2	4	6.9%	0.06 [−1.44, 1.55]
Roth AJ 2011	17.9	9.5	16	28.8	11.6	16	28.1%	−1.00 [−1.74, −0.26]
Stein MD 2012	2.4	2.5	63	2.5	2.5	56	0.0%	−0.04 [−0.40, 0.32]
W. Jihui 2020	9.44	4.96	16	13.12	5.24	14	28.0%	−0.70 [−1.45, 0.04]
Z. Lucie 2008	28.5	11.1	10	38.5	21	10	19.1%	−0.57 [−1.47, 0.33]
**Total (95% CI)**			**57**			**56**	**100.0%**	**−0.86 [−1.25, −0.46]**

Heterogeneity: $Chi^2 = 4.07$, df = 4 (P = 0.40); $I^2 = 2\%$
Test for overall effect: Z = 4.27 (P < 0.0001)

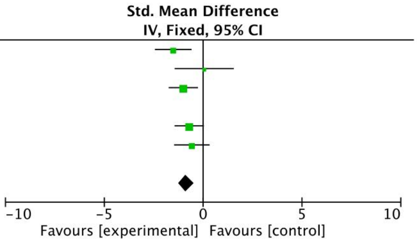

Fig. 32 Analysis on combination of treatment to N1

Study or Subgroup	Experimental Mean	Experimental SD	Experimental Total	Control Mean	Control SD	Control Total	Weight	Std. Mean Difference IV, Fixed, 95% CI
H. Kaynak 2004	3	2	12	7	3	12	9.0%	−1.51 [−2.44, −0.59]
P.M.J. Haffmans 1999	7.9	4.9	3	7.6	4.2	4	3.4%	0.06 [−1.44, 1.55]
Roth AJ 2011	17.9	9.5	16	28.8	11.6	16	14.1%	−1.00 [−1.74, −0.26]
Stein MD 2012	2.4	2.5	63	2.5	2.5	56	59.5%	−0.04 [−0.40, 0.32]
W. Jihui 2020	9.44	4.96	16	13.12	5.24	14	14.0%	−0.70 [−1.45, 0.04]
Z. Lucie 2008	28.5	11.1	10	38.5	21	10	0.0%	−0.57 [−1.47, 0.33]
**Total (95% CI)**			**110**			**102**	**100.0%**	**−0.40 [−0.67, −0.12]**

Heterogeneity: $Chi^2 = 12.93$, df = 4 (P = 0.01); $I^2 = 69\%$
Test for overall effect: Z = 2.80 (P = 0.005)

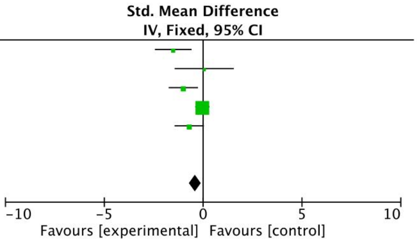

Fig. 33 subgroups analysis to N1 (treatment duration)

Study or Subgroup	Experimental Mean	Experimental SD	Experimental Total	Control Mean	Control SD	Control Total	Weight	Std. Mean Difference IV, Fixed, 95% CI
**5.1.1 1W**								
H. Kaynak 2004	3	2	12	7	3	12	8.2%	−1.51 [−2.44, −0.59]
P.M.J. Haffmans 1999	7.9	4.9	3	7.6	4.2	4	3.1%	0.06 [−1.44, 1.55]
Roth AJ 2011	17.9	9.5	16	28.8	11.6	16	12.8%	−1.00 [−1.74, −0.26]
**Subtotal (95% CI)**			**31**			**32**	**24.2%**	**−1.04 [−1.58, −0.50]**
Heterogeneity: $Chi^2 = 3.08$, df = 2 (P = 0.21); $I^2 = 35\%$								
Test for overall effect: Z = 3.77 (P = 0.0002)								
**5.1.2 ≥1M**								
Stein MD 2012	2.4	2.5	63	2.5	2.5	56	54.3%	−0.04 [−0.40, 0.32]
W. Jihui 2020	9.44	4.96	16	13.12	5.24	14	12.8%	−0.70 [−1.45, 0.04]
Z. Lucie 2008	28.5	11.1	10	38.5	21	10	8.7%	−0.57 [−1.47, 0.33]
**Subtotal (95% CI)**			**89**			**80**	**75.8%**	**−0.21 [−0.52, 0.09]**
Heterogeneity: $Chi^2 = 3.17$, df = 2 (P = 0.20); $I^2 = 37\%$								
Test for overall effect: Z = 1.37 (P = 0.17)								
**Total (95% CI)**			**120**			**112**	**100.0%**	**−0.41 [−0.68, −0.15]**

Heterogeneity: $Chi^2 = 13.06$, df = 5 (P = 0.02); $I^2 = 62\%$
Test for overall effect: Z = 3.04 (P = 0.002)
Test for subgroup differences: $Chi^2 = 6.82$, df = 1 (P = 0.009), $I^2 = 85.3\%$

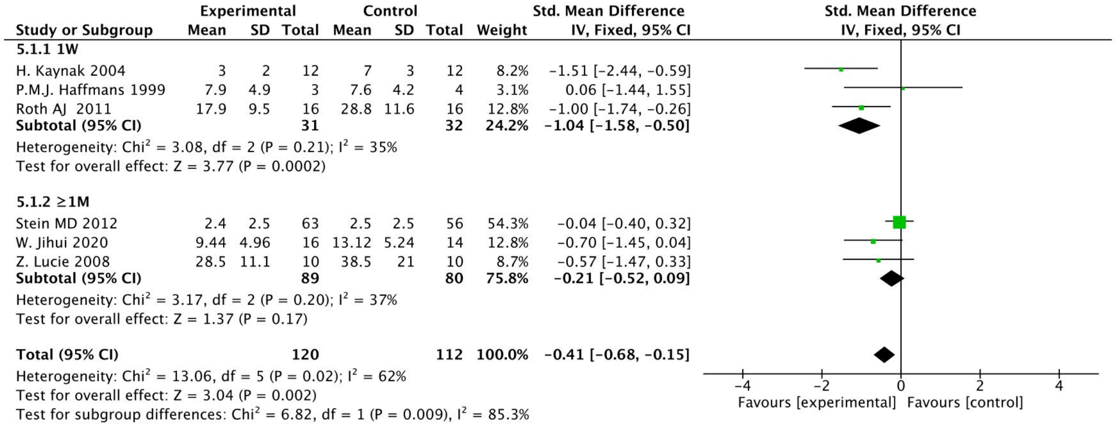

Fig. 34 subgroups analysis to N1(dosage)

Study or Subgroup	Experimental Mean	Experimental SD	Experimental Total	Control Mean	Control SD	Control Total	Weight	Std. Mean Difference IV, Random, 95% CI
**5.3.1 ≥ 100 mg/d**								
H. Kaynak 2004	3	2	12	7	3	12	14.8%	−1.51 [−2.44, −0.59]
Z. Lucie 2008	28.5	11.1	10	38.5	21	10	15.3%	−0.57 [−1.47, 0.33]
**Subtotal (95% CI)**			**22**			**22**	**30.1%**	**−1.04 [−1.96, −0.11]**
Heterogeneity: $Tau^2 = 0.23$; $Chi^2 = 2.06$, df = 1 (P = 0.15); $I^2 = 51\%$								
Test for overall effect: Z = 2.19 (P = 0.03)								
**5.3.2 ≤ 50 mg/d**								
P.M.J. Haffmans 1999	7.9	4.9	3	7.6	4.2	4	8.2%	0.06 [−1.44, 1.55]
Roth AJ 2011	17.9	9.5	16	28.8	11.6	16	18.0%	−1.00 [−1.74, −0.26]
Stein MD 2012	2.4	2.5	63	2.5	2.5	56	25.7%	−0.04 [−0.40, 0.32]
W. Jihui 2020	9.44	4.96	16	13.12	5.24	14	18.0%	−0.70 [−1.45, 0.04]
**Subtotal (95% CI)**			**98**			**90**	**69.9%**	**−0.44 [−0.98, 0.10]**
Heterogeneity: $Tau^2 = 0.16$; $Chi^2 = 6.80$, df = 3 (P = 0.08); $I^2 = 56\%$								
Test for overall effect: Z = 1.59 (P = 0.11)								
**Total (95% CI)**			**120**			**112**	**100.0%**	**−0.62 [−1.13, −0.12]**

Heterogeneity: $Tau^2 = 0.23$; $Chi^2 = 13.06$, df = 5 (P = 0.02); $I^2 = 62\%$
Test for overall effect: Z = 2.42 (P = 0.02)
Test for subgroup differences: $Chi^2 = 1.19$, df = 1 (P = 0.28), $I^2 = 15.7\%$

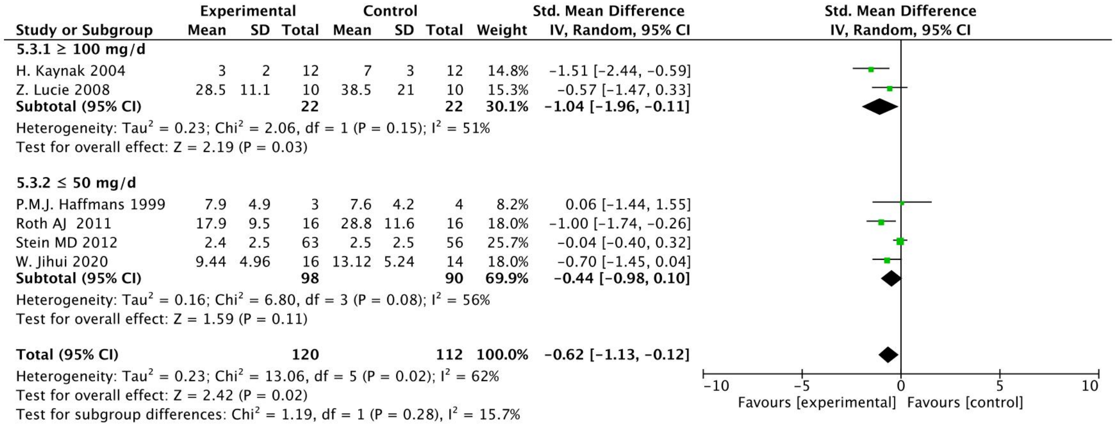

Fig. 35 Funnel plot for N1

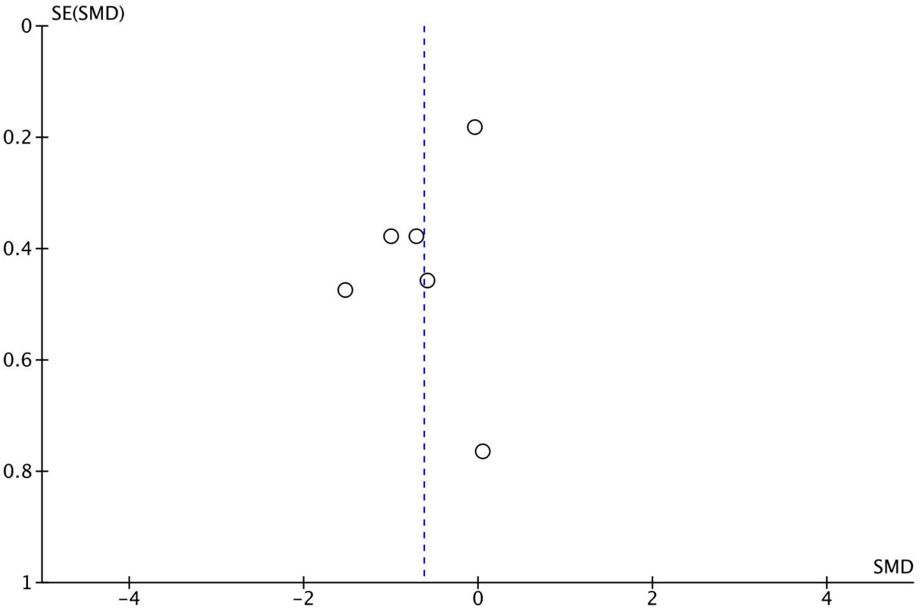

Fig. 36 pooled effects of trazodone on N2

Study or Subgroup	Experimental Mean	SD	Total	Control Mean	SD	Total	Weight	Std. Mean Difference IV, Fixed, 95% CI
H. Kaynak 2004	50	16	12	59	9	12	9.9%	−0.67 [−1.50, 0.16]
P.M.J. Haffmans 1999	41.8	13.5	3	45.7	6.9	4	2.9%	−0.33 [−1.85, 1.19]
Roth AJ 2011	223.1	47.2	16	216	58.2	16	14.1%	0.13 [−0.56, 0.82]
Stein MD 2012	63.3	13	63	63.3	13	56	52.3%	0.00 [−0.36, 0.36]
W. Jihui 2020	59.72	13.59	16	61.31	11.56	14	13.2%	−0.12 [−0.84, 0.60]
Z. Lucie 2008	148.2	60.5	10	197.4	20.9	10	7.6%	−1.04 [−1.99, −0.09]
**Total (95% CI)**			**120**			**112**	**100.0%**	**−0.15 [−0.41, 0.11]**

Heterogeneity: $Chi^2 = 6.27$, df = 5 (P = 0.28); $I^2 = 20\%$
Test for overall effect: Z = 1.15 (P = 0.25)

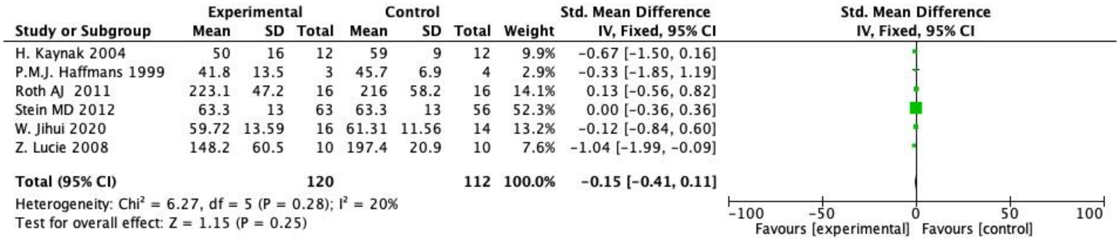

Fig. 37 Analysis on combination of treatment to N2

Study or Subgroup	Experimental Mean	SD	Total	Control Mean	SD	Total	Weight	Std. Mean Difference IV, Fixed, 95% CI
H. Kaynak 2004	0.5	0.16	12	0.59	0.09	12	10.7%	−0.67 [−1.50, 0.16]
P.M.J. Haffmans 1999	0.418	0.135	3	0.457	0.069	4	3.2%	−0.33 [−1.85, 1.19]
Roth AJ 2011	223.1	47.2	16	216	58.2	16	15.2%	0.13 [−0.56, 0.82]
Stein MD 2012	0.633	0.13	63	0.633	0.13	56	56.6%	0.00 [−0.36, 0.36]
W. Jihui 2020	0.5972	0.1359	16	0.6131	0.1156	14	14.2%	−0.12 [−0.84, 0.60]
Z. Lucie 2008	148.2	60.5	10	197.4	20.9	10	0.0%	−1.04 [−1.99, −0.09]
**Total (95% CI)**			**110**			**102**	**100.0%**	**−0.08 [−0.35, 0.19]**

Heterogeneity: $Chi^2 = 2.61$, df = 4 (P = 0.62); $I^2 = 0\%$
Test for overall effect: Z = 0.58 (P = 0.56)

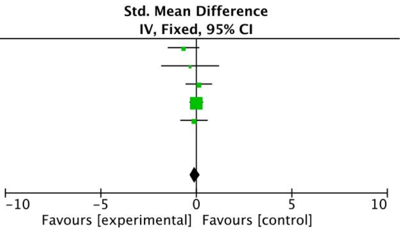

Fig. 38 subgroups analysis to N2 (treatment duration:1 week)

Study or Subgroup	Experimental Mean	SD	Total	Control Mean	SD	Total	Weight	Std. Mean Difference IV, Fixed, 95% CI
H. Kaynak 2004	50	16	12	59	9	12	36.8%	−0.67 [−1.50, 0.16]
P.M.J. Haffmans 1999	41.8	13.5	3	45.7	6.9	4	10.9%	−0.33 [−1.85, 1.19]
Roth AJ 2011	223.1	47.2	16	216	58.2	16	52.3%	0.13 [−0.56, 0.82]
Stein MD 2012	63.3	13	63	63.3	13	56	0.0%	0.00 [−0.36, 0.36]
W. Jihui 2020	59.72	13.59	16	61.31	11.56	14	0.0%	−0.12 [−0.84, 0.60]
Z. Lucie 2008	148.2	60.5	10	197.4	20.9	10	0.0%	−1.04 [−1.99, −0.09]
**Total (95% CI)**			**31**			**32**	**100.0%**	**−0.21 [−0.72, 0.29]**

Heterogeneity: $Chi^2 = 2.14$, df = 2 (P = 0.34); $I^2 = 6\%$
Test for overall effect: Z = 0.84 (P = 0.40)

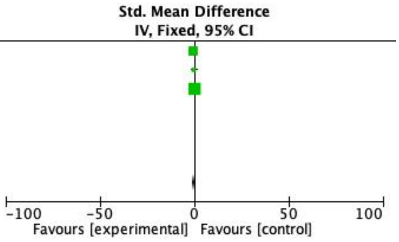

Fig. 39 subgroups analysis to N2 (treatment duration:≥1 month)

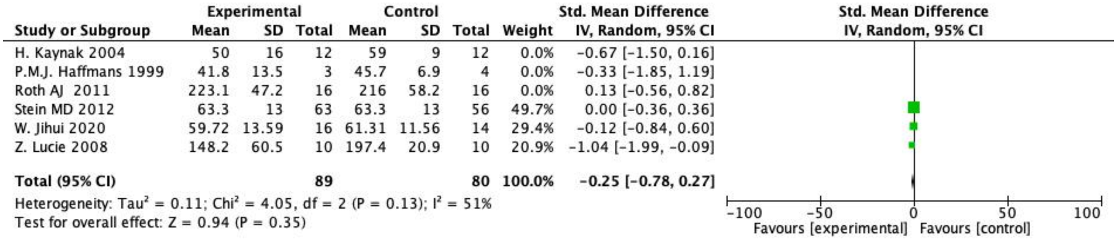

Study or Subgroup	Experimental Mean	Experimental SD	Experimental Total	Control Mean	Control SD	Control Total	Weight	Std. Mean Difference IV, Random, 95% CI
H. Kaynak 2004	50	16	12	59	9	12	0.0%	−0.67 [−1.50, 0.16]
P.M.J. Haffmans 1999	41.8	13.5	3	45.7	6.9	4	0.0%	−0.33 [−1.85, 1.19]
Roth AJ 2011	223.1	47.2	16	216	58.2	16	0.0%	0.13 [−0.56, 0.82]
Stein MD 2012	63.3	13	63	63.3	13	56	49.7%	0.00 [−0.36, 0.36]
W. Jihui 2020	59.72	13.59	16	61.31	11.56	14	29.4%	−0.12 [−0.84, 0.60]
Z. Lucie 2008	148.2	60.5	10	197.4	20.9	10	20.9%	−1.04 [−1.99, −0.09]
**Total (95% CI)**			**89**			**80**	**100.0%**	**−0.25 [−0.78, 0.27]**

Heterogeneity: $Tau^2 = 0.11$; $Chi^2 = 4.05$, df = 2 (P = 0.13); $I^2 = 51\%$
Test for overall effect: Z = 0.94 (P = 0.35)

Fig. 40 subgroups analysis to N2(dosage)

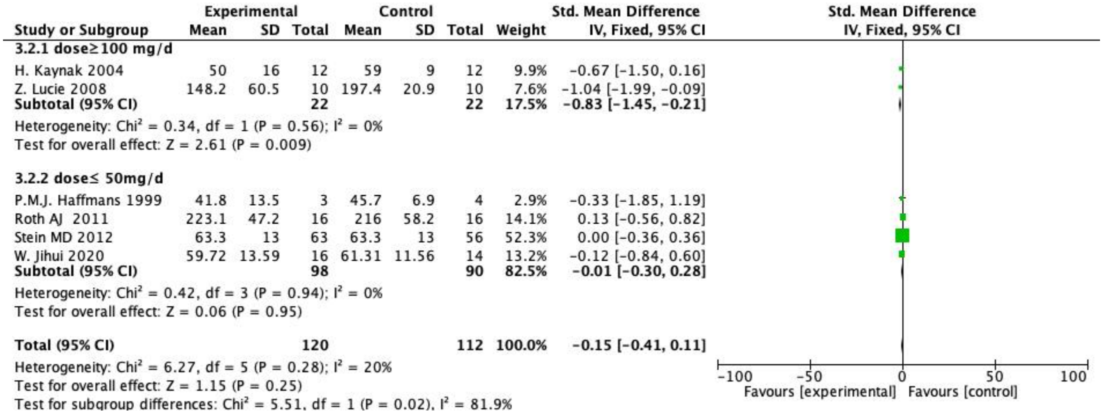

Study or Subgroup	Experimental Mean	Experimental SD	Experimental Total	Control Mean	Control SD	Control Total	Weight	Std. Mean Difference IV, Fixed, 95% CI
**3.2.1 dose≥100 mg/d**								
H. Kaynak 2004	50	16	12	59	9	12	9.9%	−0.67 [−1.50, 0.16]
Z. Lucie 2008	148.2	60.5	10	197.4	20.9	10	7.6%	−1.04 [−1.99, −0.09]
**Subtotal (95% CI)**			**22**			**22**	**17.5%**	**−0.83 [−1.45, −0.21]**
Heterogeneity: $Chi^2 = 0.34$, df = 1 (P = 0.56); $I^2 = 0\%$								
Test for overall effect: Z = 2.61 (P = 0.009)								
**3.2.2 dose≤ 50mg/d**								
P.M.J. Haffmans 1999	41.8	13.5	3	45.7	6.9	4	2.9%	−0.33 [−1.85, 1.19]
Roth AJ 2011	223.1	47.2	16	216	58.2	16	14.1%	0.13 [−0.56, 0.82]
Stein MD 2012	63.3	13	63	63.3	13	56	52.3%	0.00 [−0.36, 0.36]
W. Jihui 2020	59.72	13.59	16	61.31	11.56	14	13.2%	−0.12 [−0.84, 0.60]
**Subtotal (95% CI)**			**98**			**90**	**82.5%**	**−0.01 [−0.30, 0.28]**
Heterogeneity: $Chi^2 = 0.42$, df = 3 (P = 0.94); $I^2 = 0\%$								
Test for overall effect: Z = 0.06 (P = 0.95)								
**Total (95% CI)**			**120**			**112**	**100.0%**	**−0.15 [−0.41, 0.11]**

Heterogeneity: $Chi^2 = 6.27$, df = 5 (P = 0.28); $I^2 = 20\%$
Test for overall effect: Z = 1.15 (P = 0.25)
Test for subgroup differences: $Chi^2 = 5.51$, df = 1 (P = 0.02), $I^2 = 81.9\%$

Fig. 41 Funnel plot for N2

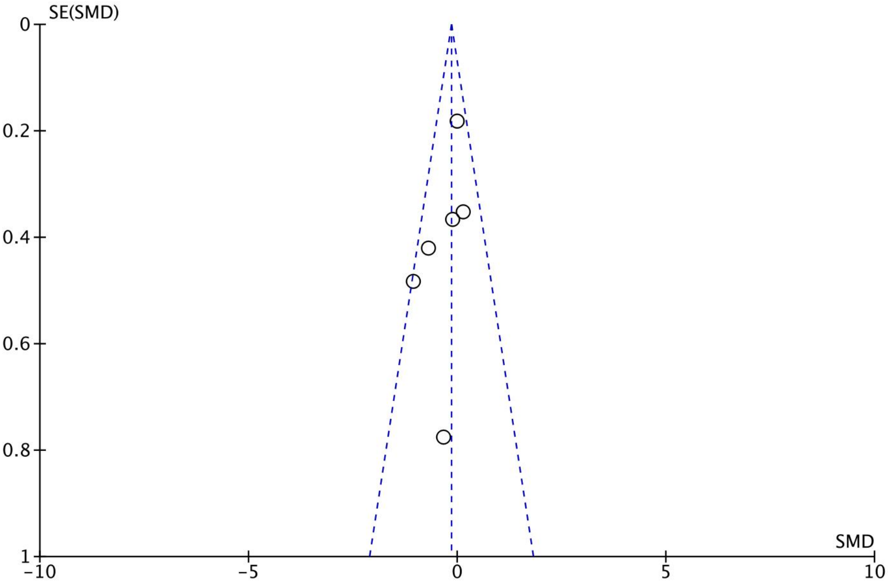

Fig. 42 pooled effects of trazodone on N3

Study or Subgroup	Experimental Mean	Experimental SD	Experimental Total	Control Mean	Control SD	Control Total	Weight	Std. Mean Difference IV, Random, 95% CI
C.-Y. Chen 2021	31.5	13.2	22	18.4	8.7	22	12.2%	1.15 [0.51, 1.79]
H. Kaynak 2004	31	13	12	19	9	12	11.6%	1.04 [0.17, 1.90]
Hongju Zhang 2013	82.02	4.96	20	42.38	1.34	18	6.5%	10.43 [7.88, 12.99]
Lanfang Cao 2018	124.04	18.09	36	79.91	16.79	37	12.2%	2.50 [1.88, 3.12]
P.M.J. Haffmans 1999	26.5	8.2	3	23.7	9.5	4	9.6%	0.26 [-1.25, 1.77]
Roth AJ 2011	90.4	33.8	16	67.9	41.4	16	12.0%	0.58 [-0.13, 1.29]
Stein MD 2012	14.5	10.3	63	14.5	10.3	56	12.7%	0.00 [-0.36, 0.36]
W. Jihui 2020	15.37	4.93	16	10.64	6.87	14	11.9%	0.78 [0.03, 1.53]
Z. Lucie 2008	120.3	61.9	10	40.2	24.9	10	11.1%	1.63 [0.58, 2.67]
**Total (95% CI)**			**198**			**189**	**100.0%**	**1.61 [0.69, 2.53]**

Heterogeneity: $Tau^2 = 1.70$; $Chi^2 = 106.94$, df = 8 ($P < 0.00001$); $I^2 = 93\%$
Test for overall effect: Z = 3.43 (P = 0.0006)

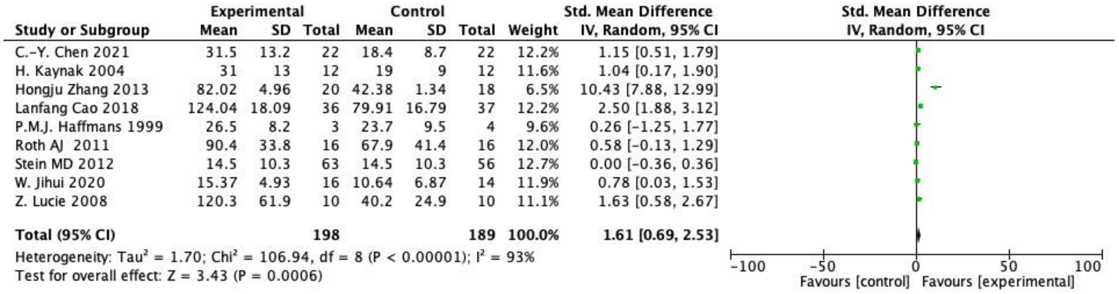

Fig. 43 subgroups analysis to N3 (mean age)

Study or Subgroup	Experimental Mean	Experimental SD	Experimental Total	Control Mean	Control SD	Control Total	Weight	Std. Mean Difference IV, Random, 95% CI
**9.3.1 >60years**								
C.-Y. Chen 2021	31.5	13.2	22	18.4	8.7	22	12.2%	1.15 [0.51, 1.79]
Lanfang Cao 2018	124.04	18.09	36	79.91	16.79	37	12.2%	2.50 [1.88, 3.12]
W. Jihui 2020	15.37	4.93	16	10.64	6.87	14	11.9%	0.78 [0.03, 1.53]
**Subtotal (95% CI)**			**74**			**73**	**36.4%**	**1.49 [0.45, 2.53]**
Heterogeneity: $Tau^2 = 0.73$; $Chi^2 = 14.63$, df = 2 (P = 0.0007); $I^2 = 86\%$								
Test for overall effect: Z = 2.80 (P = 0.005)								
**9.3.2 <60years**								
H. Kaynak 2004	31	13	12	19	9	12	11.6%	1.04 [0.17, 1.90]
Hongju Zhang 2013	82.02	4.96	20	42.38	1.34	18	6.5%	10.43 [7.88, 12.99]
P.M.J. Haffmans 1999	26.5	8.2	3	23.7	9.5	4	9.6%	0.26 [-1.25, 1.77]
Roth AJ 2011	90.4	33.8	16	67.9	41.4	16	12.0%	0.58 [-0.13, 1.29]
Stein MD 2012	14.5	10.3	63	14.5	10.3	56	12.7%	0.00 [-0.36, 0.36]
Z. Lucie 2008	120.3	61.9	10	40.2	24.9	10	11.1%	1.63 [0.58, 2.67]
**Subtotal (95% CI)**			**124**			**116**	**63.6%**	**1.80 [0.47, 3.13]**
Heterogeneity: $Tau^2 = 2.37$; $Chi^2 = 71.37$, df = 5 ($P < 0.00001$); $I^2 = 93\%$								
Test for overall effect: Z = 2.65 (P = 0.008)								
**Total (95% CI)**			**198**			**189**	**100.0%**	**1.61 [0.69, 2.53]**

Heterogeneity: $Tau^2 = 1.70$; $Chi^2 = 106.94$, df = 8 ($P < 0.00001$); $I^2 = 93\%$
Test for overall effect: Z = 3.43 (P = 0.0006)
Test for subgroup differences: $Chi^2 = 0.13$, df = 1 (P = 0.72), $I^2 = 0\%$

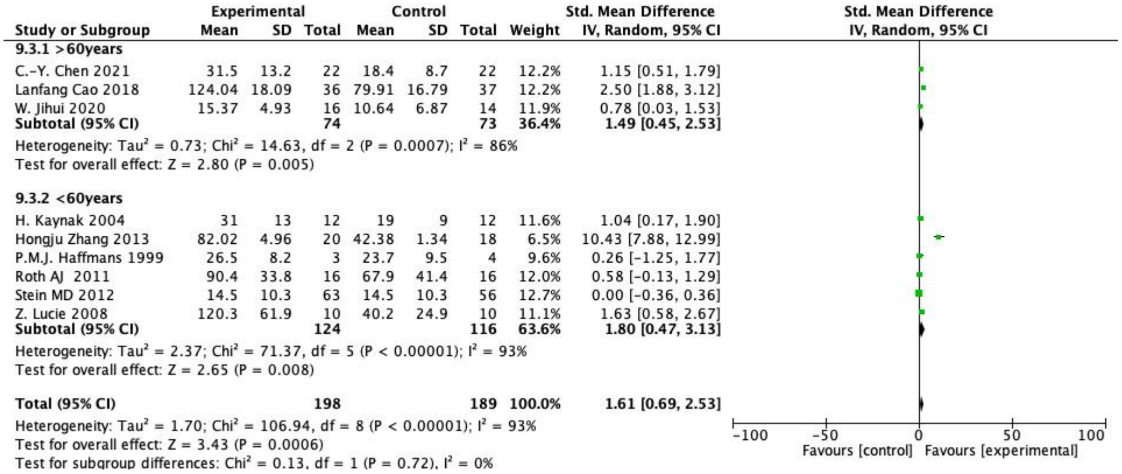

Fig. 44 subgroups analysis to N3(dosage)

Study or Subgroup	Experimental Mean	Experimental SD	Experimental Total	Control Mean	Control SD	Control Total	Weight	Std. Mean Difference IV, Random, 95% CI
**9.5.1 dose≥100 mg/d**								
C.-Y. Chen 2021	31.5	13.2	22	18.4	8.7	22	12.2%	1.15 [0.51, 1.79]
H. Kaynak 2004	31	13	12	19	9	12	11.6%	1.04 [0.17, 1.90]
Hongju Zhang 2013	82.02	4.96	20	42.38	1.34	18	6.5%	10.43 [7.88, 12.99]
Lanfang Cao 2018	124.04	18.09	36	79.91	16.79	37	12.2%	2.50 [1.88, 3.12]
Z. Lucie 2008	120.3	61.9	10	40.2	24.9	10	11.1%	1.63 [0.58, 2.67]
**Subtotal (95% CI)**			**100**			**99**	**53.7%**	**2.80 [1.31, 4.28]**
Heterogeneity: $Tau^2 = 2.50$; $Chi^2 = 55.66$, df = 4 ($P < 0.00001$); $I^2 = 93\%$								
Test for overall effect: Z = 3.69 (P = 0.0002)								
**9.5.2 dose≤ 50mg/d**								
P.M.J. Haffmans 1999	26.5	8.2	3	23.7	9.5	4	9.6%	0.26 [-1.25, 1.77]
Roth AJ 2011	90.4	33.8	16	67.9	41.4	16	12.0%	0.58 [-0.13, 1.29]
Stein MD 2012	14.5	10.3	63	14.5	10.3	56	12.7%	0.00 [-0.36, 0.36]
W. Jihui 2020	15.37	4.93	16	10.64	6.87	14	11.9%	0.78 [0.03, 1.53]
**Subtotal (95% CI)**			**98**			**90**	**46.3%**	**0.33 [-0.09, 0.75]**
Heterogeneity: $Tau^2 = 0.06$; $Chi^2 = 4.57$, df = 3 (P = 0.21); $I^2 = 34\%$								
Test for overall effect: Z = 1.52 (P = 0.13)								
**Total (95% CI)**			**198**			**189**	**100.0%**	**1.61 [0.69, 2.53]**

Heterogeneity: $Tau^2 = 1.70$; $Chi^2 = 106.94$, df = 8 ($P < 0.00001$); $I^2 = 93\%$
Test for overall effect: Z = 3.43 (P = 0.0006)
Test for subgroup differences: $Chi^2 = 9.79$, df = 1 (P = 0.002), $I^2 = 89.8\%$

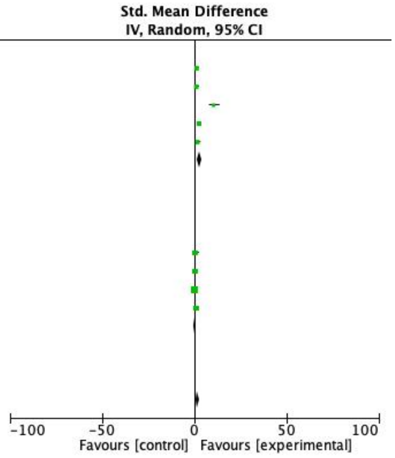

Fig. 45 subgroups analysis to N3 (treatment combination or placebo)

Study or Subgroup	Experimental Mean	SD	Total	Control Mean	SD	Total	Weight	Std. Mean Difference IV, Random, 95% CI
**6.2.1 combination with other therapies**								
Lanfang Cao 2018	124.04	18.09	36	79.91	16.79	37	12.2%	2.50 [1.88, 3.12]
Z. Lucie 2008	120.3	61.9	10	40.2	24.9	10	11.1%	1.63 [0.58, 2.67]
**Subtotal (95% CI)**			**46**			**47**	**23.4%**	**2.17 [1.33, 3.00]**
Heterogeneity: $Tau^2 = 0.19$; $Chi^2 = 2.01$, df = 1 (P = 0.16); $I^2 = 50\%$								
Test for overall effect: Z = 5.09 (P < 0.00001)								
**6.2.2 placebo**								
C.-Y. Chen 2021	31.5	13.2	22	18.4	8.7	22	12.2%	1.15 [0.51, 1.79]
H. Kaynak 2004	31	13	12	19	9	12	11.6%	1.04 [0.17, 1.90]
Hongju Zhang 2013	82.02	4.96	20	42.38	1.34	18	6.5%	10.43 [7.88, 12.99]
P.M.J. Haffmans 1999	26.5	8.2	3	23.7	9.5	4	9.6%	0.26 [-1.25, 1.77]
Roth AJ 2011	90.4	33.8	16	67.9	41.4	16	12.0%	0.58 [-0.13, 1.29]
Stein MD 2012	14.5	10.3	63	14.5	10.3	56	12.7%	0.00 [-0.36, 0.36]
W. Jihui 2020	15.37	4.93	16	10.64	6.87	14	11.9%	0.78 [0.03, 1.53]
**Subtotal (95% CI)**			**152**			**142**	**76.6%**	**1.43 [0.43, 2.44]**
Heterogeneity: $Tau^2 = 1.53$; $Chi^2 = 71.38$, df = 6 (P < 0.00001); $I^2 = 92\%$								
Test for overall effect: Z = 2.80 (P = 0.005)								
**Total (95% CI)**			**198**			**189**	**100.0%**	**1.61 [0.69, 2.53]**
Heterogeneity: $Tau^2 = 1.70$; $Chi^2 = 106.94$, df = 8 (P < 0.00001); $I^2 = 93\%$								
Test for overall effect: Z = 3.43 (P = 0.0006)								
Test for subgroup differences: $Chi^2 = 1.21$, df = 1 (P = 0.27), $I^2 = 17.6\%$								

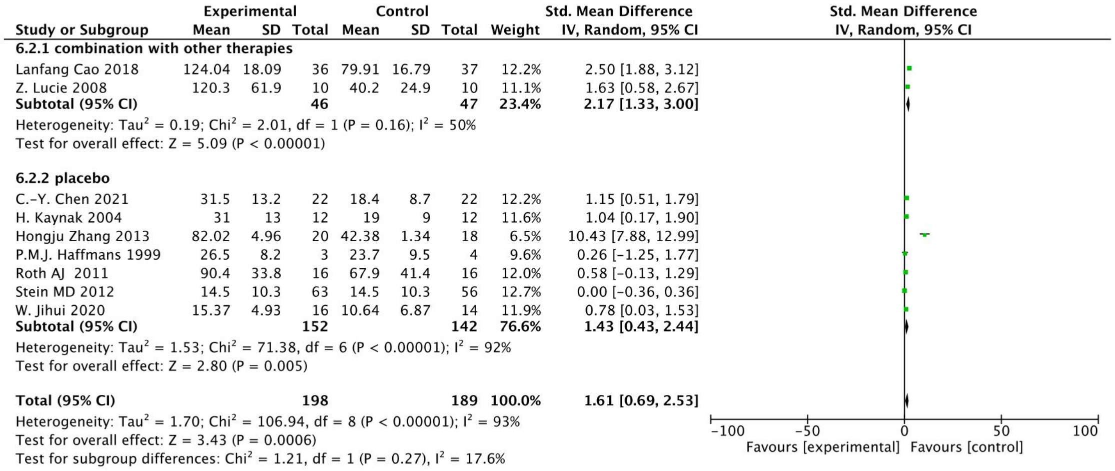

Fig. 46 subgroups analysis to N3 (treatment duration)

Study or Subgroup	Experimental Mean	SD	Total	Control Mean	SD	Total	Weight	Std. Mean Difference IV, Random, 95% CI
**9.4.1 1W**								
C.-Y. Chen 2021	31.5	13.2	22	18.4	8.7	22	10.9%	1.15 [0.51, 1.79]
H. Kaynak 2004	31	13	12	19	9	12	10.4%	1.04 [0.17, 1.90]
Hongju Zhang 2013	52.09	2.83	20	43.51	2.34	18	10.1%	3.22 [2.22, 4.21]
P.M.J. Haffmans 1999	26.5	8.2	3	23.7	9.5	4	8.8%	0.26 [-1.25, 1.77]
Roth AJ 2011	90.4	33.8	16	67.9	41.4	16	10.8%	0.58 [-0.13, 1.29]
**Subtotal (95% CI)**			**73**			**72**	**51.0%**	**1.27 [0.39, 2.16]**
Heterogeneity: $Tau^2 = 0.79$; $Chi^2 = 20.38$, df = 4 (P = 0.0004); $I^2 = 80\%$								
Test for overall effect: Z = 2.82 (P = 0.005)								
**9.4.2 ≥1M**								
Hongju Zhang 2013	82.02	4.96	20	42.38	1.34	18	6.1%	10.43 [7.88, 12.99]
Lanfang Cao 2018	124.04	18.09	36	79.91	16.79	37	10.9%	2.50 [1.88, 3.12]
Stein MD 2012	14.5	10.3	63	14.5	10.3	56	11.3%	0.00 [-0.36, 0.36]
W. Jihui 2020	15.37	4.93	16	10.64	6.87	14	10.7%	0.78 [0.03, 1.53]
Z. Lucie 2008	120.3	61.9	10	40.2	24.9	10	10.0%	1.63 [0.58, 2.67]
**Subtotal (95% CI)**			**145**			**135**	**49.0%**	**2.57 [0.88, 4.26]**
Heterogeneity: $Tau^2 = 3.35$; $Chi^2 = 104.77$, df = 4 (P < 0.00001); $I^2 = 96\%$								
Test for overall effect: Z = 2.99 (P = 0.003)								
**Total (95% CI)**			**218**			**207**	**100.0%**	**1.80 [0.88, 2.71]**
Heterogeneity: $Tau^2 = 1.89$; $Chi^2 = 127.66$, df = 9 (P < 0.00001); $I^2 = 93\%$								
Test for overall effect: Z = 3.85 (P = 0.0001)								
Test for subgroup differences: $Chi^2 = 1.79$, df = 1 (P = 0.18), $I^2 = 44.0\%$								

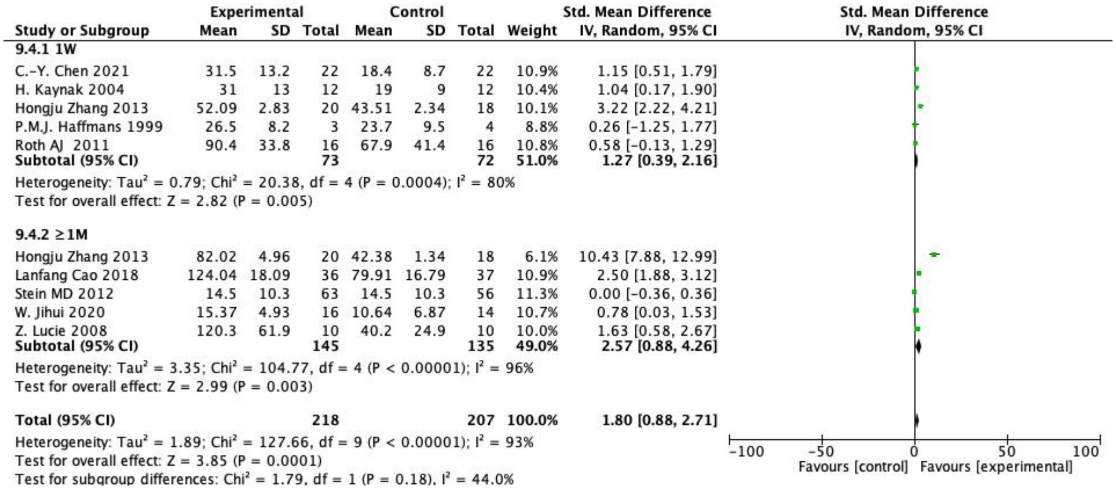

Fig. 47 Funnel plot for N3

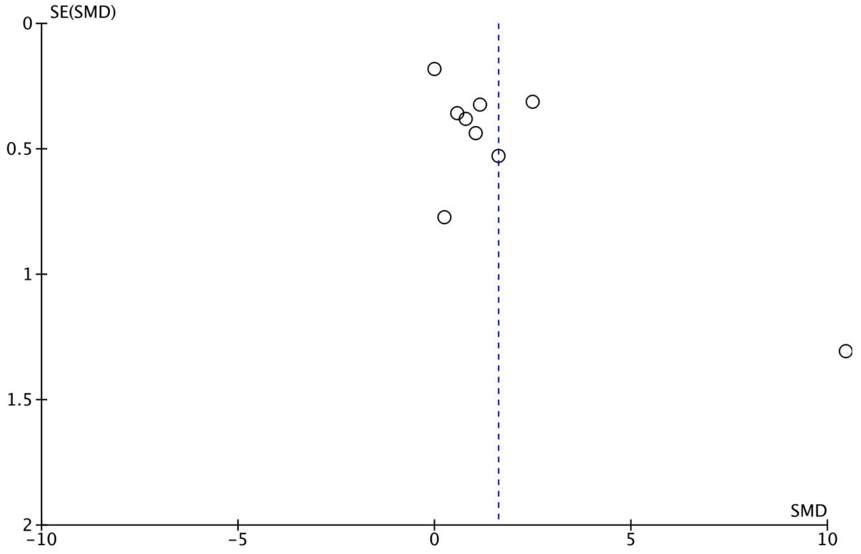

Fig. 48 discontinuation for all causes

Study or Subgroup	Experimental Events	Experimental Total	Control Events	Control Total	Weight	Odds Ratio M-H, Fixed, 95% CI
C.-Y. Chen 2021	2	24	2	24	7.5%	1.00 [0.13, 7.75]
H. Kaynak 2004	0	12	0	12		Not estimable
Hongju Zhang 2013	0	20	2	20	10.0%	0.18 [0.01, 4.01]
Lanfang Cao 2018	0	36	0	37		Not estimable
Le Bon O 2003	1	9	1	9	3.6%	1.00 [0.05, 18.91]
P.M.J. Haffmans 1999	0	3	0	4		Not estimable
Roth AJ 2011	0	16	0	16		Not estimable
Stein MD 2012	6	69	12	68	45.2%	0.44 [0.16, 1.26]
W. Jihui 2020	4	20	6	20	19.6%	0.58 [0.14, 2.50]
Xuemin Li 2021	5	36	4	36	14.1%	1.29 [0.32, 5.26]
Z. Lucie 2008	0	10	0	10		Not estimable
**Total (95% CI)**		**255**		**256**	**100.0%**	**0.63 [0.33, 1.18]**
Total events	18		27			

Heterogeneity: $Chi^2 = 2.36, df = 5 (P = 0.80); I^2 = 0\%$
Test for overall effect: Z = 1.44 (P = 0.15)

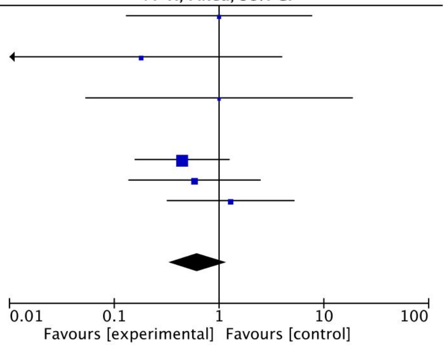

Fig. 49 Adverse event: dizziness

Study or Subgroup	trazodone Events	trazodone Total	Control Events	Control Total	Weight	Odds Ratio M-H, Fixed, 95% CI
C.-Y. Chen 2021	0	24	0	24		Not estimable
H. Kaynak 2004	0	12	0	12		Not estimable
Hongju Zhang 2013	0	20	0	20		Not estimable
Lanfang Cao 2018	0	36	0	37		Not estimable
Le Bon O 2003	2	9	0	9	3.7%	6.33 [0.26, 152.86]
P.M.J. Haffmans 1999	0	3	0	4		Not estimable
Roth AJ 2011	0	16	0	16		Not estimable
Stein MD 2012	16	67	7	67	53.2%	2.69 [1.03, 7.05]
W. Jihui 2020	3	20	4	20	33.9%	0.71 [0.14, 3.66]
Xuemin Li 2021	2	31	1	32	9.2%	2.14 [0.18, 24.86]
Z. Lucie 2008	0	10	0	10		Not estimable
**Total (95% CI)**		**248**		**251**	**100.0%**	**2.10 [1.00, 4.41]**
Total events	23		12			

Heterogeneity: $Chi^2 = 2.40, df = 3 (P = 0.49); I^2 = 0\%$
Test for overall effect: Z = 1.97 (P = 0.05)

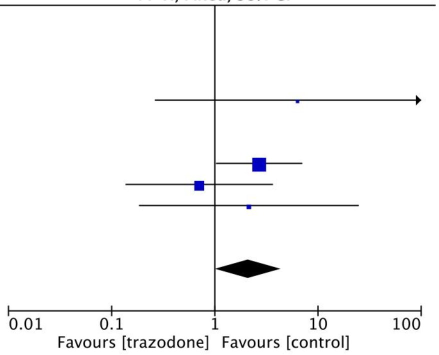

Fig. 50 Adverse event: headache

Study or Subgroup	trazodone Events	trazodone Total	Control Events	Control Total	Weight	Odds Ratio M-H, Fixed, 95% CI
C.-Y. Chen 2021	0	24	0	24		Not estimable
H. Kaynak 2004	0	12	0	12		Not estimable
Hongju Zhang 2013	0	20	0	20		Not estimable
Lanfang Cao 2018	0	36	0	37		Not estimable
Le Bon O 2003	0	9	2	9	22.5%	0.16 [0.01, 3.81]
P.M.J. Haffmans 1999	0	3	0	4		Not estimable
Roth AJ 2011	0	16	0	16		Not estimable
Stein MD 2012	8	67	4	67	33.4%	2.14 [0.61, 7.47]
W. Jihui 2020	1	20	2	20	18.0%	0.47 [0.04, 5.69]
Xuemin Li 2021	2	31	3	32	26.2%	0.67 [0.10, 4.29]
Z. Lucie 2008	0	10	0	10		Not estimable
**Total (95% CI)**		**248**		**251**	**100.0%**	**1.01 [0.43, 2.36]**
Total events	11		11			

Heterogeneity: $Chi^2 = 3.23, df = 3 (P = 0.36); I^2 = 7\%$
Test for overall effect: Z = 0.02 (P = 0.99)

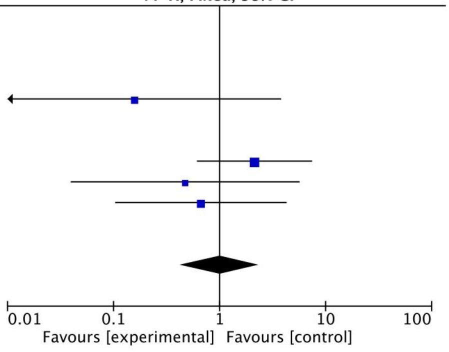

Fig. 51 Adverse event: daytime drowsiness

Study or Subgroup	trazodone Events	trazodone Total	Control Events	Control Total	Weight	Odds Ratio M-H, Fixed, 95% CI
C.-Y. Chen 2021	0	24	0	24		Not estimable
H. Kaynak 2004	2	12	0	12	5.2%	5.95 [0.26, 138.25]
Hongju Zhang 2013	0	20	0	20		Not estimable
Lanfang Cao 2018	0	36	0	37		Not estimable
Le Bon O 2003	5	9	1	9	5.7%	10.00 [0.85, 117.02]
P.M.J. Haffmans 1999	0	3	0	4		Not estimable
Roth AJ 2011	0	16	0	16		Not estimable
Stein MD 2012	15	67	9	67	89.2%	1.86 [0.75, 4.61]
W. Jihui 2020	0	20	0	20		Not estimable
Z. Lucie 2008	0	10	0	10		Not estimable
**Total (95% CI)**		**217**		**219**	**100.0%**	**2.53 [1.14, 5.64]**
Total events	22		10			

Heterogeneity: $Chi^2 = 1.93$, df = 2 (P = 0.38); $I^2 = 0\%$
Test for overall effect: Z = 2.27 (P = 0.02)

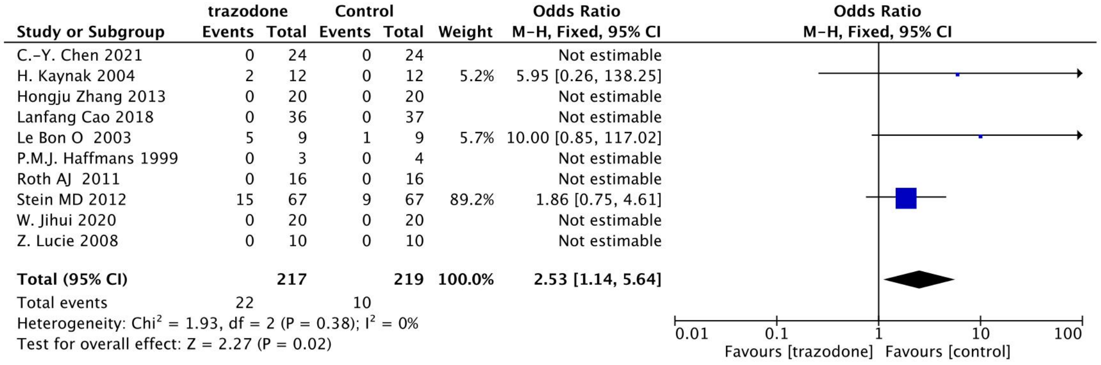

Fig. 52 Adverse event: decreased appetite

Study or Subgroup	trazodone Events	trazodone Total	Control Events	Control Total	Weight	Odds Ratio M-H, Fixed, 95% CI
C.-Y. Chen 2021	0	24	0	24		Not estimable
H. Kaynak 2004	1	12	0	12	7.3%	3.26 [0.12, 88.35]
Hongju Zhang 2013	0	20	0	20		Not estimable
Lanfang Cao 2018	0	36	0	37		Not estimable
Le Bon O 2003	0	9	0	9		Not estimable
P.M.J. Haffmans 1999	0	3	0	4		Not estimable
Roth AJ 2011	0	16	0	16		Not estimable
Stein MD 2012	13	67	5	67	66.4%	2.99 [1.00, 8.91]
W. Jihui 2020	4	20	2	20	26.3%	2.25 [0.36, 13.97]
Z. Lucie 2008	0	10	0	10		Not estimable
**Total (95% CI)**		**217**		**219**	**100.0%**	**2.81 [1.14, 6.92]**
Total events	18		7			

Heterogeneity: $Chi^2 = 0.08$, df = 2 (P = 0.96); $I^2 = 0\%$
Test for overall effect: Z = 2.25 (P = 0.02)

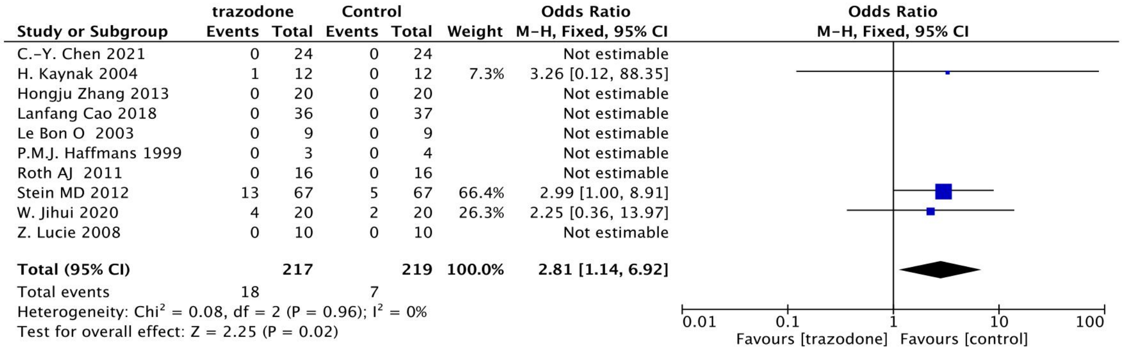

Article title: Trazodone Changed the Polysomnographic Sleep Architecture in Insomnia: A Systematic Review and Meta-Analysis

journal name: Drugs

author names: Yongliang Zheng[1] • Tian Lv[2] • Jingjing Wu[3] • Yumeng Lyu[1]

Affiliation:

1 School of Rehabilitation Medicine, Jiangsu Vocational College of Medicine, Yancheng, China

2 Department of Neurology, Zhuji Hospital Affiliated Shaoxing University, Shaoxing, China

3 Department of Cardiology, Pudong New Area People's Hospital, Shanghai, China

The corresponding author and e-mail address:

Yongliang Zheng, 12131@jsmc.edu.cn
