# Supplementary material for: Trazodone changed the polysomnographic sleep architecture in insomnia disorder: a systematic review and meta-analysis
Source: Sci Rep. 2022 Aug 24;12:14453. doi: 10.1038/s41598-022-18776-7 (PMC9402537; doi:10.1038/s41598-022-18776-7)

Assessment of the risk of bias by the Revised Cochrane risk-of-bias tool for randomized trials (RoB 2) for each outcome
(Fig. 1-7: TST, LPS, WASO, N1, N3, REM, AHI)

Fig. 1 Assessment of the risk of bias for TST

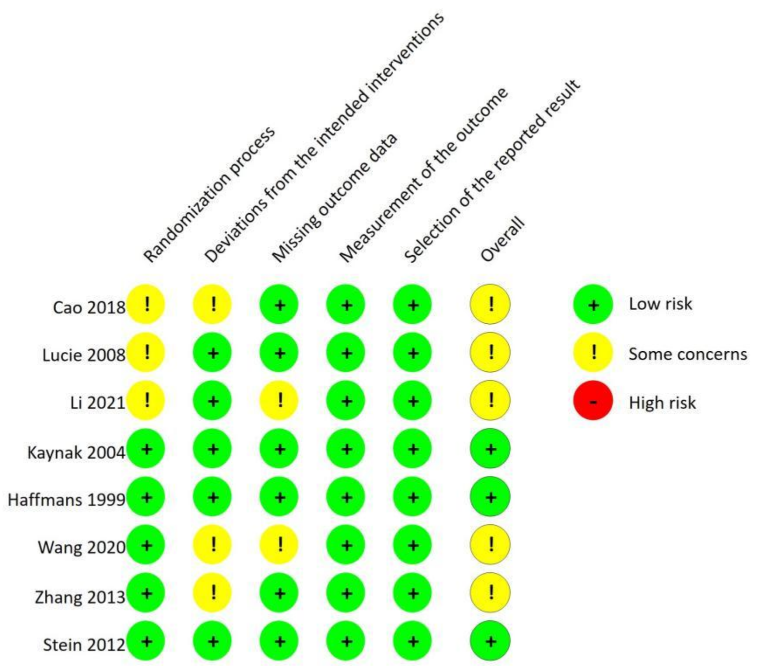

Fig. 2 Assessment of the risk of bias for LPS

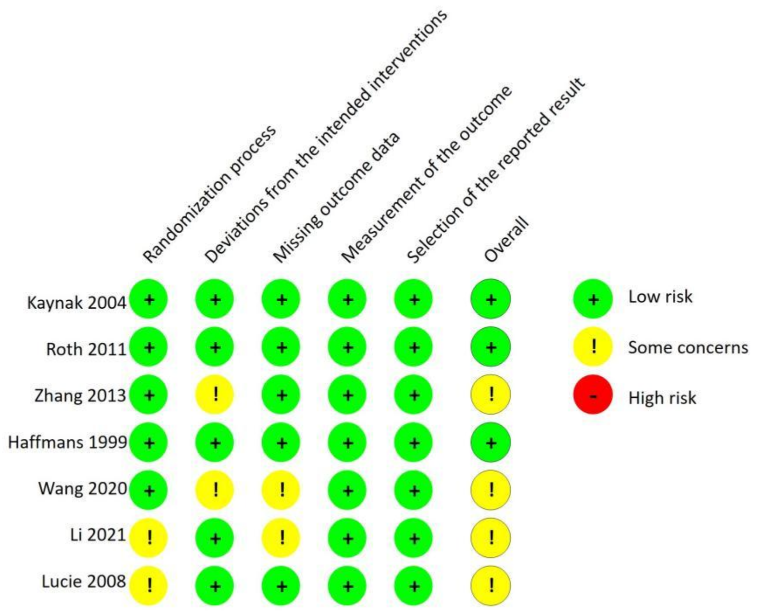

Fig. 3 Assessment of the risk of bias for WASO

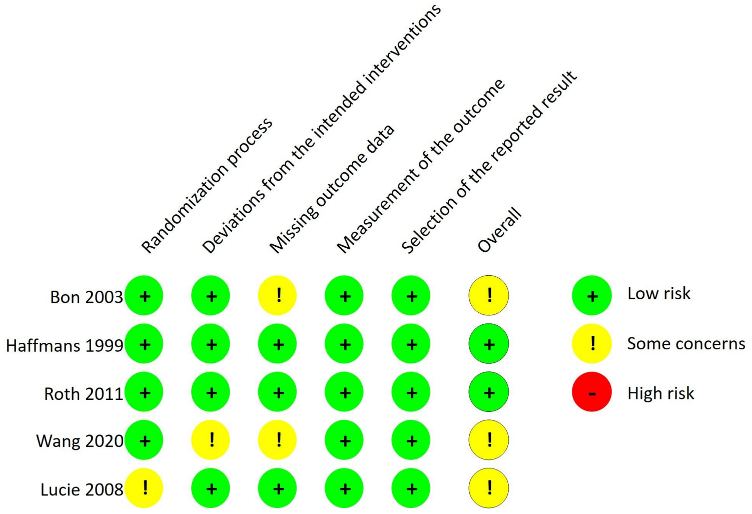

Fig. 4 Assessment of the risk of bias for N1

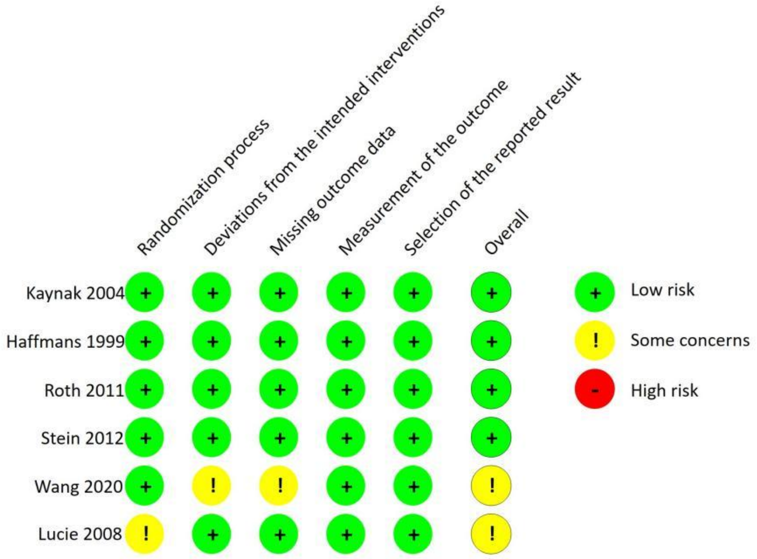

Fig. 5 Assessment of the risk of bias for N3

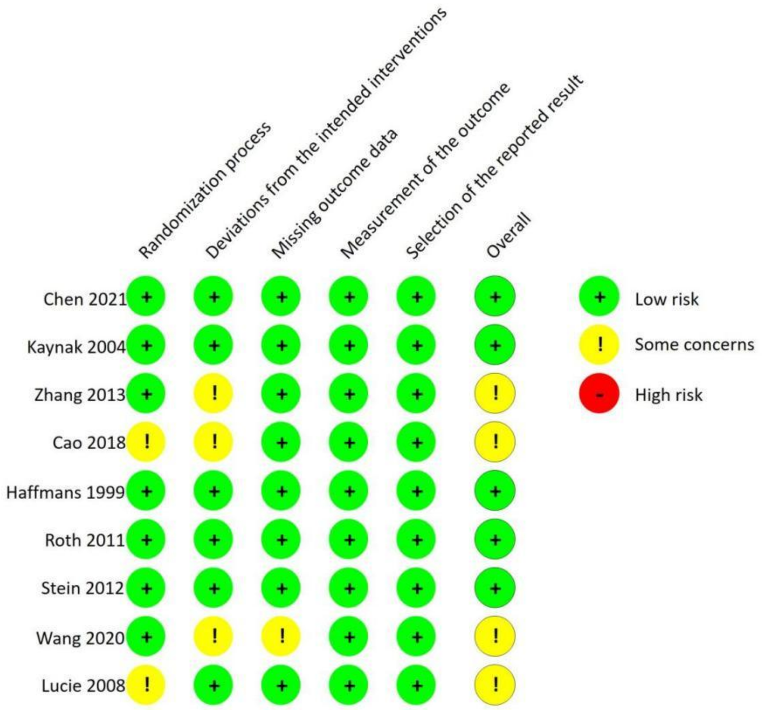

Fig. 6 Assessment of the risk of bias for REM

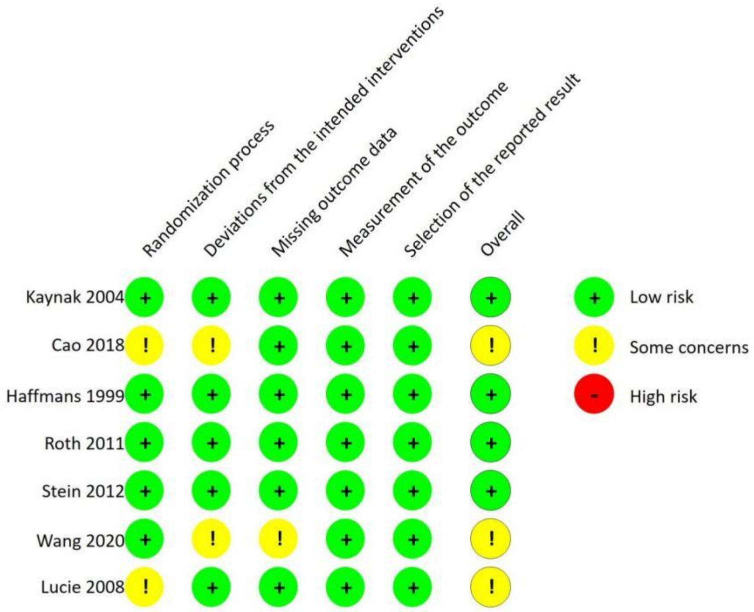

Fig. 7 Assessment of the risk of bias for AHI

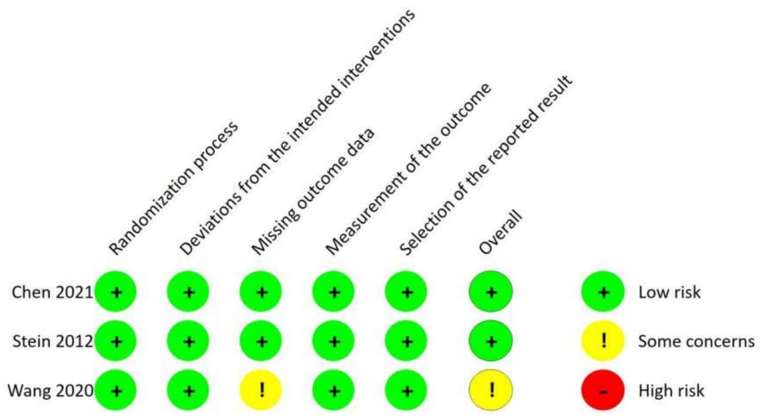

Fig. 8 Assessment of the risk of bias for NAs

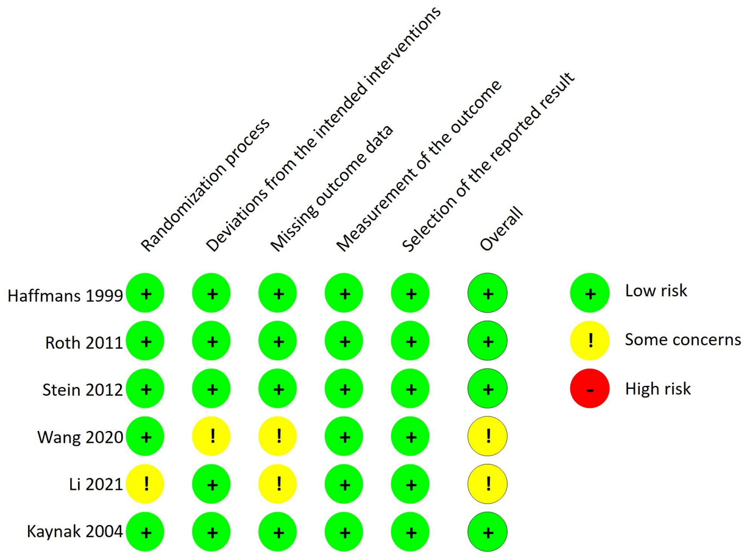

Supplement: Supplementary file 4 — Supplementary Information 4. [file 41598_2022_18776_MOESM4_ESM.pdf]
